# Interventions Using Wearable Physical Activity Trackers Among Adults With Cardiometabolic Conditions: A Systematic Review and Meta-analysis

**DOI:** 10.1001/jamanetworkopen.2021.16382

**Published:** 2021-07-20

**Authors:** Alexander Hodkinson, Evangelos Kontopantelis, Charles Adeniji, Harm van Marwijk, Brian McMillian, Peter Bower, Maria Panagioti

**Affiliations:** 1 National Institute for Health Research, School for Primary Care Research, Manchester Academic Health Science Centre, University of Manchester, Manchester, United Kingdom; 2 Department of Primary Care and Public Health, Brighton and Sussex Medical School, University of Brighton, Brighton, United Kingdom

## Abstract

**Importance:**

Wearable physical activity (PA) trackers, such as accelerometers, fitness trackers, and pedometers, are accessible technologies that may encourage increased PA levels in line with current recommendations. However, whether their use is associated with improvements in PA levels in participants who experience 1 or more cardiometabolic conditions, such as diabetes, prediabetes, obesity, and cardiovascular disease, is unknown.

**Objective:**

To assess the association of interventions using wearable PA trackers (accelerometers, fitness trackers, and pedometers) with PA levels and other health outcomes in adults with cardiometabolic conditions.

**Data Sources:**

For this systematic review and meta-analysis, searches of MEDLINE, Embase, Cochrane Central Register of Controlled Trials, and PsycINFO were performed from January 1, 2000, until December 31, 2020, with no language restriction. A combination of Medical Subject Heading terms and text words of *diabetes*, *obesity, cardiovascular disease, pedometers, accelerometers,* and *Fitbits* were used.

**Study Selection:**

Randomized clinical trials or cluster randomized clinical trials that evaluated the use of wearable PA trackers, such as pedometers, accelerometers, or fitness trackers, were included. Trials were excluded if they assessed the trackers only as measuring tools of PA before and after another intervention, they required participants to be hospitalized, assessors were not blinded to the trackers, or they used a tracker to measure the effect of a pharmacological treatment on PA among individuals.

**Data Extraction and Synthesis:**

The study followed the Preferred Reporting Items for Systematic Reviews and Meta-analyses (PRISMA) reporting guideline. A random-effects model was used for the meta-analysis.

**Main Outcomes and Measures:**

The primary outcome was mean difference in PA levels. When the scale was different across studies, standardized mean differences were calculated. Heterogeneity was quantified using the *I*^2^ statistic and explored using mixed-effects metaregression.

**Results:**

A total of 38 randomized clinical trials with 4203 participants were eligible in the systematic review; 29 trials evaluated pedometers, and 9 evaluated accelerometers or fitness trackers. Four studies did not provide amenable outcome data, leaving 34 trials (3793 participants) for the meta-analysis. Intervention vs comparator analysis showed a significant association of wearable tracker use with increased PA levels overall (standardized mean difference, 0.72; 95% CI, 0.46-0.97; *I*^2^ = 88%; 95% CI, 84.3%-90.8%; *P* < .001) in studies with short to medium follow-up for median of 15 (range, 12-52) weeks. Multivariable metaregression showed an association between increased PA levels and interventions that involved face-to-face consultations with facilitators (23 studies; β = −0.04; 95% CI, −0.11 to −0.01), included men (23 studies; β = 0.48; 95% CI, 0.01-0.96), and assessed pedometer-based interventions (26 studies; β = 0.20; 95% CI, 0.02-0.32).

**Conclusions and Relevance:**

In this systematic review and meta-analysis, interventions that combined wearable activity trackers with health professional consultations were associated with significant improvements in PA levels among people with cardiometabolic conditions.

## Key Points

**Question:**

Are interventions using wearable physical activity (PA) trackers, including accelerometers, fitness trackers, and/or pedometers, associated with improved PA levels among people with cardiometabolic conditions?

**Findings:**

In this systematic review and meta-analysis of 38 randomized clinical trials with 4203 participants, interventions with wearable PA trackers were associated with significantly increased PA levels during approximately 15-weeks follow-up; interventions (particularly pedometers) with additional components, such as consultations with health care professionals, were associated with increased PA levels.

**Meaning:**

The findings suggest that use of wearable PA trackers (especially pedometers) is associated with increased PA levels among people with cardiometabolic conditions when combined with additional intervention components, such as face-to-face consultations, but these PA level improvements may remain below the targets set by clinical recommendations.

## Teaser

This systematic review an meta-analysis assesses the association of interventions using wearable physical activity tracking devices with physical activity levels and other health outcomes among adults with cardiometabolic conditions.

## Introduction

A large proportion of the population experiences cardiometabolic conditions, such as type 2 diabetes, prediabetes states (eg, obesity), and cardiovascular disease.^1,2^ In the UK, 3.3 million people have been diagnosed with type 2 diabetes, and most of them experience additional cardiometabolic conditions or risks, including obesity, increased blood pressure, disturbed blood lipid levels, and a tendency to develop thrombosis and cardiovascular disease.^3^ The increasing prevalence of cardiometabolic conditions combined with demographic changes mean that the overall cardiometabolic costs will account for more than 20% of the entire National Health Service budget in the next 20 years, with most of these costs being avoidable.^4^

Despite the health and economic consequences of cardiometabolic conditions, they are mainly lifestyle related and can be improved by targeting unhealthy lifestyle behaviors. Particularly, a low physical activity (PA) level is a fundamental modifiable risk behavior for people with cardiometabolic conditions and is a major opportunity for intervention.^5^ Premature deaths could potentially be prevented by addressing low levels of PA more so than any other risk factor,^6^ such as smoking, alcohol consumption, or stress-related illnesses. Recognizing the importance of PA, several public health guidelines recommend reaching and maintaining health-enhancing levels of PA^7^ and promote PA interventions in the community and the workplace.^8,9,10^ However, promoting PA for people with cardiometabolic conditions remains a challenge.^11^

Wearable activity trackers may empower people with cardiometabolic conditions to improve their PA levels and health behaviors. These devices include simple activity trackers, such as pedometers (a portable electrical or electromechanical tracker that counts each step a person takes by detecting the motion of the person along the body’s long axis)^12^ and accelerometers or fitness trackers (electromechanical trackers used to measure acceleration forces by the use of algorithms to accurately detect periods of wear and nonwear time).^13^ These wearable activity trackers have recently become popular for motivating people to be more active and monitoring that activity among people with a range of chronic conditions, including cardiometabolic conditions.^14^ The devices are simple, relatively affordable, user-friendly, and potentially motivational.^15^

A number of previous systematic reviews^16,17,18,19^ suggest that these wearable trackers may be associated with increased PA levels among people with chronic conditions, including cardiometabolic conditions. However, there is limited evidence on whether interventions that involve wearable activity trackers are associated with improved PA levels among people with cardiometabolic conditions in the short and long terms. Moreover, to our knowledge, factors that may moderate the effectiveness of these interventions (eg, intervention or patient factors) have not been examined using robust methods, such as metaregression. These knowledge gaps are major barriers for the wider use of these monitoring devices in the care of people with cardiometabolic conditions.

Our earlier systematic review^20^ was retracted based on a complex issue that involved the intervention definition, which affected the inclusion criteria for 9 of the 36 studies included in the original systematic review. Thus, in this updated systematic review and meta-analysis, we examined whether interventions that use activity trackers (pedometers, accelerometers, or fitness trackers) as part of the program design were associated with short-term and long-term improvements in PA levels and health outcomes, including blood glucose levels, blood pressure, cholesterol levels, body weight, and body mass index (BMI; calculated as weight in kilograms divided by height in meters squared), among people with cardiometabolic conditions compared with usual care. Metaregression was also used to examine whether an association with increased PA levels in the intervention group vs comparator group was moderated by the characteristics of the interventions (type of wearable tracker, setting daily goals, use of consultations with facilitators, evaluation length, use of a theoretical framework, and uptake rate) and patients (age, sex, and index condition).

## Methods

This systematic review and meta-analysis (CRD42018104448) was conducted and reported in accordance with the Cochrane Handbook^21^ and the Preferred Reporting Items for Systematic Reviews and Meta-analyses (PRISMA) reporting guideline.^22^ Searches were performed in the Cochrane Central Register of Controlled Trials (CENTRAL), Embase, MEDLINE and PsycINFO from January 1, 2000, until December 31, 2020, with no language restriction. January 2000 was used because an earlier review^16^ reported that no studies were found before this date. A combination of Medical Subject Heading terms and text words of *diabetes*, *obesity, cardiovascular disease, pedometers, accelerometers,* and *Fitbits* were used. The full search strategy in MEDLINE is available in eTable 1 in the Supplement. Additional studies were obtained from screening the reference lists of included trials and previous systematic reviews. Experts were contacted in the field to inquire about unpublished studies. Trial registers (ClinicalTrials.gov, ISCTRN, the World Health Organization International Clinical Trials Registry Platform, and OpenTrials.net) were also searched to identify any unpublished or ongoing trials.

### Eligibility Criteria

#### Population

Adults 18 years or older with a diagnosis of type 2 diabetes (or at risk for type 2 diabetes), obesity or overweight, and cardiovascular disease were eligible for inclusion. For obesity classification, the World Health Organization definition was used to standardize across studies.^23^ Studies of people diagnosed with stroke or who had undergone surgery were excluded.

#### Intervention

Randomized clinical trials (RCTs) or cluster RCTs that evaluated the use of wearable activity trackers, such as pedometers, accelerometers, or fitness trackers, were part of the intervention program. Excluded trials were those that used the wearable trackers only as measuring tools of PA before and after another intervention (and thus were not part of the interventional program design), those that required participants to be hospitalized, those in which assessors were not blinded to the wearable trackers, and those that used a wearable tracker to measure the effect of a pharmacologic treatment on an individual’s ability to be physically active.

#### Comparator and Outcome

Participants using wearable activity trackers were compared with a group receiving usual care. The primary outcome was the association of the use of an activity tracker with PA levels. Secondary outcomes were body weight or BMI, blood glucose level, blood pressure, and cholesterol levels.

### Data Collection and Extraction

Titles and abstracts were assessed by 3 reviewers (A.H., M.P., and C.A.). Data extraction was conducted by 1 reviewer (A.H.) and checked by a second (C.A.) for consistency. A modified version of the Cochrane Public Health Group’s data extraction template^24^ was used after pilot testing on 5 studies to ensure reliability. The Oxford Implementation Index was used to assess implementation of the intervention and contextual factors.^25^ This index was adapted for the purposes of this review.

### Assessment of Risk of Bias

Risk of bias for each study was assessed by 2 reviewers (A.H., C.A.) using the original version of the Cochrane Risk of Bias tool.^26^ The blinding of participants and personnel was not included in the risk of bias assessment because many studies did not report this domain since it was impossible to blind participants while they were using the technology device. If further information was required on any aspect of study design or outcome, related publications and trial protocols were sought and authors were contacted. Adjustment for cluster RCTs was performed in accordance with the Cochrane Handbook (§23.1.4).^21^

### Missing Data

Study authors were contacted by email where there were missing or unclear data (for instance relating to the primary outcome). Studies for which insufficient primary data were available (eg, missing data cannot be obtained) were excluded from the meta-analysis but not from the review.

### Statistical Analysis

The statistical analysis proceeded in 2 stages. First, Hartung-Knapp-Sidik-Jonkman random-effects meta-analyses^27^ were conducted to assess the association of the interventions with the primary and secondary outcomes compared with controls. The Hartung-Knapp-Sidik-Jonkman method was used instead of the DerSimonian-Laird random-effects method because it is globally thought to be a more robust method of choice when study sizes are small and considerable heterogeneity is present. Because all outcomes were continuous, standardized mean differences (SMDs) were calculated using Hedges *g*.^28^ Standardized mean differences were interpreted according to the Cohen rule of interpretation in accordance with the Cochrane guidelines (ie, small effect, 0.2; moderate effect, 0.5; and large effect, 0.8).^29,30^ When required, data were transformed to SMDs using Comprehensive Meta-analysis software.^31^ Physical activity outcomes were objectively classified by intervention measure (ie, daily step count or moderate to vigorous physical activity [MVPA]). Pooled associations with 95% CIs are presented, and forest plots with *I*^2^ and the test-based 95% CIs^32^ are used to show statistical heterogeneity among studies. When a study contributed more than 1 intervention arm to the analysis, the arms were combined to avoid double counting of the control group. When data were judged to be insufficient to include in meta-analyses, the results were synthesized narratively.

Second, mixed-effects univariable and multivariable (multilevel) metaregression analyses examined the association of several study-level covariates with the primary outcome (PA levels). The multilevel aspect of the regression model allowed for potential clustering by including random effects for tracker type and study. Ten covariates were selected based on importance and consultation among the authors. Then the categories for the covariates were coded using consensus procedures and as informed by the Oxford Implementation Index^25^: (1) sex classified by dominance in each study (ie, mostly male or female), (2) age (<50 vs ≥50 years), (3) index condition (ie, type 2 diabetes, overweight or obesity, or cardiovascular disease), (4) type of tracker, (5) outcome measure used to assess PA (steps per day or MVPA), (6) consultations with facilitators, (7) intervention length (≤4 vs >4 months), (8) goal set for physical activity, (9) theory-based intervention, and (10) intervention uptake. Low risk of bias based on all 5 domains that were judged to be low risk was assessed against the high-risk studies with at least 1 high-risk domain in a sensitivity analysis. Covariates that met our significance criterion (2-sided *P* < .15) were entered into a multivariable metaregression model. The *P* < .15 threshold was conservative to avoid prematurely discounting potentially important explanatory variables, and adjusted tests were used for controlling type I error.^33^ All analyses were performed using the *meta* and *metafor* packages in R, version 4.0.3 (R Foundation for Statistical Computing). For each meta-analysis with 10 studies or more, funnel plots, the Begg test, and the Egger test were used to examine publication bias. The trim-and-fill method was used as a sensitivity analysis to observe possible small study publication bias.

## Results

**Figure 1.**
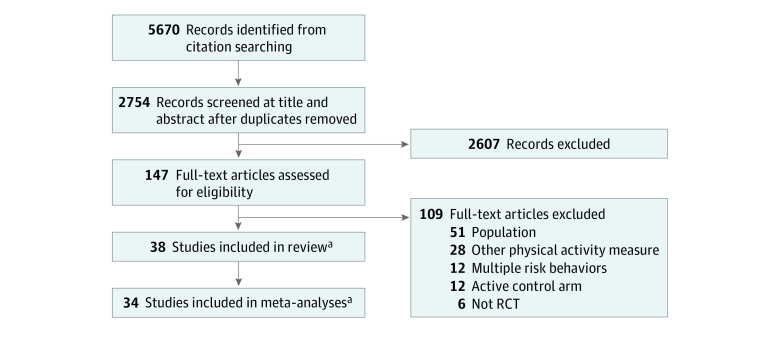
Flow Diagram of Screening Stages RCT indicates randomized clinical trial. ^a^No unpublished studies were found.

After duplicates were removed, the search retrieved 5670 references. After abstract and title screening of 2754 references and full-text screening of 147 studies, 38 studies^34,35,36,37,38,39,40,41,42,43,44,45,46,47,48,49,50,51,52,53,54,55,56,57,58,59,60,61,62,63,64,65,66,67,68,69,70,71,72,73,74,75,76,77,78,79,80,81,82^ met our inclusion criteria (Figure 1). All eligible studies are listed in eTable 2 in the Supplement. No unpublished studies were identified.

### Characteristics of Included Studies

#### Location, Setting, and Participant Characteristics

Most studies^34,35,36,37,38,39,41,42,43,45,76,82^ were conducted in the US (n = 12) or UK (n = 6). The settings of the studies varied and included hospitals, primary care practices, medical and community centers, and universities. The 38 studies^34,35,36,37,38,39,40,41,42,43,44,45,46,47,48,49,50,51,52,53,54,55,56,57,58,59,60,61,62,63,64,65,66,67,68,69,70,71,72,73,74,75,76,77,78,79,80,81,82^ involved 4203 participants (Table 1). All the studies included adults with a mean age between 35 and 67 years; 16 studies^38,42,43,50,51,53,54,65,66,67,69,71,72,75,78,79^ focused on older adults with a mean age older than 60 years. Two studies^34,35^ included women only, and the remainder^36,37,38,39,40,41,42,43,44,45,46,47,48,49,50,51,52,53,54,55,56,57,58,59,60,61,62,63,64,65,66,67,68,69,70,71,72,73,74,75,76,77,78,79,80,81,82^ involved mixed-sex populations. The target population recruited in the studies was predominantly those diagnosed with type 2 diabetes (n = 23), but other diagnoses included cardiovascular diseases (n = 8) and obesity or mixed obesity and overweight (n = 7).

**Table 1.** Participant Characteristics From Eligible Studies

Source	Participants in, No.	Location	Age, mean (SD), y	Sex, No. (%)	Race/ethnicity	Target population for study recruitment	Multimorbidity or other health issues at baseline
**Pedometer**							
Alonso-Dominguez et al,^66^ 2019	204	Spain	60.8 (7.8)^a^	52 (51%) Female	100% White	Type 2 diabetes	56% Hypertensive; 59% dyslipidaemia; 29.5 (4.2) BMI
Anderson et al,^34^ 2015	38	US	57 (10.8)	Intervention: 3 of 18 female (17%); control: 8 of 20 female (40%)	57% White	Coronary artery disease	No
Andrews et al,^46^ 2011	345	UK	Intervention: 60 (9.7); control: 59.5 (11.1)	Intervention: 66% male; control: 63% male	Intervention: 96% White; control: 97% White	Newly diagnosed type 2 diabetes	No
Araiza et al,^67^ 2006	30	Mexico	Intervention: 49 (11);control: 51 (10)	NR	NR	Type 2 diabetes diagnosed	No
Bjorgaas et al,^57^ 2008	48	Norway	Intervention: 56.4 (11); control: 61.2 (9.7)	Intervention: 9 female, 14 male; control: 8 female, 17 male	NR	Type 2 diabetes diagnosed (age <80 y)	Intervention (6 of 23) and control (8 of 25): other metabolic disease(s) but were none reported
Chudowolska-Kielkowska et al,^68^ 2020	199	Poland	Intervention: 62 (7); control: 63 (7)	Intervention: 29 male (34%); control: 26 male (33%)	100% White	Cardiovascular risk factors	Obesity; hypertension; diabetes; dyslipidaemia
Cupples et al,^69^ 2012	45	UK	Intervention: 61.6 (11.3); control: 59.2 (8.9)	91% Male	NR	Participants in cardiac rehabilitation	No
Dasgupta et al,^70^ 2017	347	Canada	Intervention: 60 (11.2); control: 59.4 (11.4)	Intervention: 56.9% female; control: 52.6% female	Intervention: 63.6% White; control: 57% White	Type 2 diabetes and/or hypertension	No
De Greef et al,^64^ 2010	41	Belgium	NR	68% Male	NR	Type 2 diabetes diagnosed during 6 mo	No
De Greef et al,^71^ 2011	67	Belgium	Overall: 62 (IQR, 9)	69% Male	NR	Type 2 diabetes diagnosed	No
Van Dyck and De Greef,^72^ 2011	47	Belgium	Overall: 67.4 (9.3)	70.1% Male; 29.9% female	NR	Type 2 diabetes diagnosed	No
Diedrich et al,^35^ 2010	32	US	Intervention: 56.68 (13.62); control: 54.88 (9.79)	NR	NR	Type 2 diabetes diagnosed	No
Engel et al,^53^ 2006	50	Australia	Intervention: 60.5 (7.34); control: 64 (6.76)	Intervention: 13 male, 11 female; control: 15 male, 15 female	NR	Type 2 diabetes diagnosed	High number of participants with obesity included
Fayehun et al,^74^ 2018	46	Nigeria	NR	63% Female; 37% male	91.3% Yoruba, 8.7% others	Type 2 diabetes diagnosed	No
Greaney et al,^36^ 2017	181	US	Intervention: 36.62 (5.07); usual care: 35.62 (5.76)	100% Female	100% Black	Overweight or obesity (Black women with low SES)	No
Grey et al,^48^ 2019	60	UK	Intervention: 50.3 (8.9); control: 49.5 (9.1)	Intervention: 57% male; control: 55% male	Intervention: 90% White, 3% Black, 3% Asian, other 3%; control: 100% White	Adults with overweight or obesity	No
Houle et al,^55^ 2011; Houle et al,^74^ 2012	65	Canada	Intervention: 58 (8); control: 59 (9)	14 of 65 Female (21.5%)	NR	Acute coronary syndrome	No
Katzmarzyk et al,^37^ 2011	43	US	Intervention: 52.7 (8.8); control: 50.3 (7.7)	Intervention: 20% male; control: 13% male	Intervention: 70% White; control: 73.9% White	Overweight or obesity (BMI, 25-35)	No
Kirk et al,^49^ 2009	127	UK	Intervention 1: 60.9 (9.6); intervention 2: 63.2 (10.6); usual care: 59.2 (10.4)	Intervention 1: 53% male and 47% female; intervention 2: 42% male and 58% female; usual care: 51% male and 49% female	NA	Type 2 diabetes	No
Lewis et al,^38^ 2020	40	US	Intervention: 63.2 (5.7); control: 64 (5.1)	Intervention: 65% female; control: 85%	Intervention: 70% White, 10% Hispanic, 15% Black/African American, 5% other; control: 60% White, 15% Hispanic, 20% Black/African American, 5% other	Overweight	No
Paula et al,^75^ 2015	40	Brazil	Intervention: 61.8 (8.1); control: 62.5 (8.8)	Intervention: 12 male and 8 female; control: 6 male and 14 female	Intervention: 80% White; control: 90% White	Type 2 diabetes	Yes
Pekmezi et al,^39^ 2017	76	US	Overall: 57 (4.7)	100% Female	100% African American	Overweight and obesity	No
Piette et al,^76^ 2011	339	US	56 (10.1)	51.5% Female	White: 84%; Black: 9%; other: 7%	Type 2 diabetes and depressive symptoms	Yes
Plotnikoff et al,^65^ 2013	287	Canada	Group 1: 61 (11.7); group 2: 61.4 (12.6); group 3: 62.3 (11.1)	Group 1: 46.8% female; group 2: 40.6% female; group 3: 51% female	NR	Type 2 diabetes	Yes
Silfee et al,^41^ 2016	24	US	Intervention: 57.75 (9.818); control: 57.09 (9.093)	Intervention: 75% female; control: 63.6% female	Intervention: 58.3% White, 33.3% Black, 8.3% other; control: 90.9% White, 9.1% Black	Type 2 diabetes and overweight or obesity	Yes
Tudor-Locke et al,^77^ 2004	47	Canada	Overall: 52.7 (5.2)	26 Male; 11 female	NR	Type 2 diabetes diagnosed (mean [SD] BMI, 33.3 [5.6])	No
Van Dyck et al,^78^ 2013	92	Belgium	Overall: 62 (9)	69% Male	NR	Type 2 diabetes diagnosed at age >5 y and mean (SD) BMI of 30 (2.8)	Yes
Yates et al,^50^ 2009	87	UK	65 (8)	66% Male	75% White, 24% South Asian, 1% Black	Overweight and obesity with impaired glucose tolerance	Yes
Yates et al,^51^ 2017	571	UK	Overall: 62.6 (8.2)	65.5% Male	86.8% White European	Individuals 18-74 y of age were included if they scored >90th percentile on the risk calculator (a noninvasive risk calculator for risk of developing type 2 diabetes)	Unclear
**Accelerometer and fitness trackers**							
Claes et al,^54^ 2020	120	European Union (multiple sites)	Intervention: 61.7 (14.5); control: 59.6 (13.2)	Intervention: 49 male and 11 female; control: 49 male and 11 female	100% White	Secondary prevention for CVDs	Yes
Frederix et al,^79^ 2015	139	Belgium	61 (9)	25 of 139 Female (18%)	No	Coronary artery disease or heart failure	Yes
Guiraud et al,^80^ 2012	29	France	57.4 (12.4)	5 of 29 Female (17%)	NR	Coronary artery disease or heart failure	Yes
Karstoft et al,^81^ 2013	32	Denmark	Interval walking: 57.5 (2.4); continuous walking: 60.8 (2.2); control: 57.1 (3)	Interval walking: 7 male and 5 female; continuous walking: 8 male and 4 female; control: 5 male and 3 female	NR	Type 2 diabetes	Yes
Lyons et al,^42^ 2017	40	US	61.48 (5.60)	85% Female	65% White, 13% Black, 15% other	Overweight or obesity	Yes
Lystrup et al,^43^ 2020	120	US	Intervention: 64 (9); control: 63 (7)	Intervention: 59.6% male; control: 50% male	Intervention: 53.9% White, 23.1% Black; control: 51.8% White, 21.4% Black	Type 2 diabetes	No
Martin et al,^82^ 2015	48	US	58 (8)	54% Male	79% White	CVD rehabilitation	Yes
Miyamoto et al,^56^ 2017	31	Japan	LPA: 61.7 (1.9); N-LPA: 60 (3.1); control: 60.2 (3)	LPA: 9 male and 2 female; N-LPA: 9 male and 3 female; control: 8 male and 2 female	NA	>1 y After diagnosis of type 2 diabetes	No
Paschali et al,^45^ 2005	26	US	Intervention: 48.8 (6.1); control: 47 (7.2)	53% Female in each group	NR	Obesity and type 2 diabetes	No

Abbreviations: BMI, body mass index (calculated as weight in kilograms divided by height in meters squared); CVD, cardiovascular disease; LPA, locomotive physical activity; NA, not applicable; N-LPA, nonlocomotive physical activity; NR, not reported; SES, socioeconomic status.

^a^ Median (interquartile range).

#### Intervention and Outcome Characteristics

Twenty-nine studies^34,35,36,37,38,39,41,46,48,49,50,51,53,55,57,65,66,67,68,69,70,71,72,73,74,75,76,77,78^ involved pedometers as part of the intervention program to encourage PA, whereas 9 studies^42,43,45,54,56,79,80,81,82^ used accelerometers or fitness trackers as part of the intervention program to encourage PA. In terms of content, interventions focused mainly on the association with PA levels, prevention of disease, and weight management (eTable 3 in the Supplement). Seventeen studies^36,38,39,40,41,48,49,50,51,55,64,65,71,74,76,77,78,82^ (42%) used a theoretical framework that consisted of social cognitive approaches (eg, health belief model, theory of planned behavior, or transtheoretical model); however, behavior change outcomes were not reported in the results of 11 studies.^36,38,41,46,48,49,51,54,71,72,76,77^ Twelve studies^34,36,37,39,42,50,51,54,56,70,73,82^ tested simple pedometer, accelerometer, or fitness tracker interventions. This finding meant, for instance, that after an initial consultation session, patients were provided with the accelerometer, fitness tracker, or pedometer and a log book to self-monitor their outcomes using written instructions, but no additional support was provided by facilitators or health care professionals. A total of 26 studies^35,38,41,43,45,46,48,49,53,55,57,64,65,66,67,68,69,71,72,74,75,76,77,78,79,80,81^ (61%) tested interventions that also involved consultation sessions (ie, patients were supported by facilitators who were mainly health care professionals via face-to-face consultations and/or regular telecommunications during the intervention). The median duration for receiving the intervention was 17 weeks, but this varied considerably across studies (range, 1 week to 18 months). Physical activity was measured using steps per day in 25 studies^34,37,41,42,43,48,50,51,55,65,66,67,68,69,70,71,72,73,74,75,76,77,78,81,82^ (66%) and MVPA in 9 studies^36,38,39,45,46,49,54,56,80^ (24%).

#### Risk of Bias

The quality of the studies was variable (eTable 4 in the Supplement). A total of 22 studies^37,39,42,45,46,49,50,51,54,55,56,66,68,69,70,71,72,73,74,76,78,79,82^ (58%) had a low risk of bias for the random sequence generation, and 20 studies^37,41,42,43,46,49,50,51,54,60,64,65,68,69,71,72,75,76,78^ (53%) had low risk for allocation concealment. Only 1 study^36^ was deemed high risk for this criterion. Similarly, blinding of outcome assessment was moderately reported, with 16 studies^49,51,53,55,57,66,68,69,70,71,72,74,78,79,81,82^ (42%) reporting low risk; however, 12 studies^34,35,38,41,42,43,45,46,48,54,73,76^ (32%) reported high risk for this domain. Criteria for incomplete outcome data were mostly satisfied across studies, displaying low risk in 27 studies^34,37,38,42,43,46,48,49,50,51,53,54,55,56,66,69,70,71,72,74,75,76,77,78,79,80,82^ (70%); however, 8 studies^35,36,41,57,65,69,73,77^ (21%) reported high risk. For selective reporting, only 3 studies^37,38,39^ (8%) exhibited high risk of bias.

### Synthesis of Results

Thirty-four of the 38 studies^34,36,37,38,39,40,41,42,43,44,45,46,47,48,49,50,51,52,54,55,56,58,59,60,61,62,63,64,65,66,67,68,69,70,71,72,73,74,75,76,77,78,80,81,82^ (89%) were included in the meta-analysis, involving 3793 participants. The exclusion of 4 studies^35,53,57,79^ was because the studies were not amenable for inclusion. The results of those studies are reported narratively in the Narrative Synthesis subsection of the Results section.

#### PA Levels

Summary estimates from the meta-analyses are presented in Table 2. Across all studies^34,36,37,38,39,40,41,42,43,44,45,46,47,48,49,50,51,52,54,55,56,58,59,60,61,62,63,64,65,66,67,68,69,70,71,72,73,74,75,76,77,78,80,81,82^ that involved interventions with wearable activity trackers vs comparators, there was a significant association between wearable tracker use and increased PA during an approximately 15-week period (SMD, 0.72; 95% CI, 0.46-0.97; prediction interval, −0.72 to 2.16; *I*^2^ = 88%; 95% CI, 84.3%-90.8%; *P* < .001) (eFigure 1 in the Supplement). Pedometer-based interventions were significantly associated with increased PA compared with comparators (SMD, 0.68; 95% CI, 0.44-0.93). Accelerometer- and fitness tracker–based interventions were not associated with increased PA compared with comparators (SMD, 0.92; 95% CI, −0.10 to 1.94). For pedometer-based interventions, the PA measure translated to 1877.30 steps per day (95% CI, 1139.70-2614.90 steps per day) in the intervention group compared with the usual care group (eFigure 2 in the Supplement). This value is generally lower than recommendations of governments and agencies globally,^61^ with a mean of 3000 steps being achieved daily (National Obesity Forum UK: 3000-6000 steps per day as sedentary; America on the Move: an additional 2000 steps each day to stop weight gain). Heterogeneity was high for both interventions encompassing pedometer use (*I*^2^ = 89%; 95% CI, 85%-92%) and accelerometer or fitness tracker use (*I*^2^ = 86%; 95% CI, 74%-92%).

**Table 2.** Meta-analysis of the Association of Accelerometer, Fitness Tracker, and Pedometer Interventions With Physical Activity Levels

Outcome	Trials, No.	Participants, No.	Program length, median, wk	Random-effects meta-analysis^a^					
				MD (95% CI)	*I*^2^ (test-based 95% CI)	*P* value	SMD (95% CI)	*I*^2^ (test-based 95% CI)	*P* value
Physical activity intervention									
Interventions combined	34	3793	15	NA	NA	NA	0.72 (0.46 to 0.97)	88 (84.3 to 90.8)	<.001
Accelerometer and fitness tracker	8	414	12	NA	NA	NA	0.92 (−0.10 to 1.94)	86 (74.4 to 92.3)	<.60^b^
Pedometer	26	3379	15	1877.30 (1139.70 to 2614.90)	96 (95 to 96.8)	<.001	0.68 (0.44 to 0.93)	89 (85.1 to 91.9)	NA
Outcome measure									
Steps per day	25	2893	15	NA	NA	NA	0.85 (0.53 to 1.17)	90 (86.5 to 92.6)	.008^b^
MVPA	9	900	12	NA	NA	NA	0.30 (0.00 to 0.61)	47 (0 to 75.4)	NA
Secondary outcome measures									
Glucose level, %	26	2069	24	−0.14 (−0.27 to −0.01)	80 (71.4 to 86)	.04	NA	NA	NA
Accelerometer and fitness tracker	5	415	26	0.00 (−0.24 to 0.23)	0 (0 to 79.2)	.10^b^	NA	NA	NA
Pedometer	13	1654	26	−0.19 (−0.35 to −0.03)	85 (75.9 to 90.7)	NA	NA	NA	NA
Blood pressure, mm Hg									
Systolic	21	2166	16	−0.54 (−2.90 to 1.81)	32 (0 to 60)	.44^b^	NA	NA	NA
Diastolic	20	2073	16	−2.05 (−5.39 to 1.29)	86 (79.7 to 90.3)	NA	NA	NA	NA
Cholesterol, mg/dL									
Total	14	1523	26	NA	NA	NA	−0.07 (−0.27 to 0.13)	57 (22 to 76.3)	.54^b^
High-density lipoprotein	11	1444	26	NA	NA	NA	−0.07 (−0.26 to 0.11)	51 (2.4 to 75.4)	NA
Low-density lipoprotein	10	1295	24	NA	NA	NA	0.05 (−0.15 to 0.24)	39 (0 to 70.9)	NA
BMI	19	1734	13	−0.38 (−1.20 to 0.44)	59 (32 to 75.3)	.34	NA	NA	NA
Accelerometer and fitness tracker	7	529	12	0.35 (−1.63 to 2.33)	72 (39.4 to 87.1)	.22^b^	NA	NA	NA
Pedometer	10	1205	13	−0.74 (−1.54 to 0.06)	50 (0 to 75.8)	NA	NA	NA	NA
Weight, kg	17	1758	16	0.13 (−2.70 to 2.96)	52 (16.5 to 72.4)	.92	NA	NA	NA
Accelerometer and fitness tracker	7	515	18	1.99 (−1.97 to 5.96)	23 (0 to 65.8)	.20^b^	NA	NA	NA
Pedometer	10	1243	16	−1.26 (−5.70 to 3.19)	55 (8.4 to 77.9)	NA	NA	NA	NA

Abbreviations: BMI, body mass index (calculated as weight in kilograms divided by height in meters squared); MD, mean difference; MVPA, moderate-vigorous physical activity; NA, not applicable; SMD, standardized mean difference.

SI conversion factors: To convert cholesterol to millimoles per liter, multiply by 0.0259.

^a^ Hartung-Knapp random-effects meta-analysis was applied for all outcomes.

^b^ *P* value are for the test for subgroup differences; otherwise the *P* values are for the test for heterogeneity in meta-analysis with no subgroups.

The cumulative plot (eFigure 3 in the Supplement) of PA performance based on total session time engagement provided no evidence that studies with longer periods of engagement in PA performed better in general. However, 9 studies^38,43,44,48,50,69,70,76,81^ did not report the length of the sessions and therefore could not be included.

#### Moderators of Associations

The results of the univariable and multivariable analyses are given in Table 3. Interventions using consultations with a health care professional (β = 0.45; 95% CI, 0.001-0.93; *P* = .04), pedometer-based interventions (β = 0.29; 95% CI, 0.02-0.38; *P* = .03), steps per day used as outcome measurement (β = 0.48; 95% CI, 0.04-0.76; *P* = .04), and the inclusion of predominately male participants in studies (β = 0.19; 95% CI, 0.03-0.29; *P* = .02) were associated with improved PA levels in the univariable regression analyses. The remaining factors, including index diagnosis of participants, age of participants, length of the intervention, goal setting, underpinning the intervention with a theoretical framework, intervention uptake, and risk of bias scores, were not associated with PA level and were not eligible for inclusion in the multivariable regression analysis. The overall multivariable model was statistically significant (χ^2^_3_ = 24.18; *P* = .03), and the *I*^2^ statistic decreased from 68% to 39%. Consultations with a health care professional (*b* = −0.04; 95% CI, −0.11 to −0.01; *P* = .01), pedometer use (*b* = 0.20; 95% CI, 0.02-0.32; *P* = .004), and male sex (*b* = 0.48; 95% CI, 0.01-0.96, *P* = .046) remained significantly associated with increased PA in the multivariable model. Thus, interventions that involved regular consultations with health care professionals (compared with self-monitoring only), male sex, and pedometer-based interventions (compared with accelerometers and fitness trackers) were the 3 main factors associated with improved PA levels.

**Table 3.** Univariable and Multivariable Metaregressions for PA Tracker Use

Covariate of interest	*b* (95% CI)	*P* value	*I*^2^, %	*R*^2^, %^a^
Univariable				
Type of tracker: pedometer vs accelerometer or fitness tracker	0.29 (0.02 to 0.38)	.03	51.73	10.57
Outcome measure: steps per day vs MVPA	0.48 (0.04 to 0.76)	.04	45.45	15.52
Consultations with facilitators (delivery): facilitated delivery vs self-reported	0.45 (0.001 to 0.93)	.04	35.94	20.44
Index condition: type 2 diabetes vs overweight or obese or cardiovascular disease	0.10 (−0.37 to 0.56)	.68	43.46	5.27
Sex: male vs female dominant	0.19 (0.03 to 0.29)	.02	43.05	0.58
Age: <50 vs ≥50 y	−0.20 (−0.84 to 0.44)	.52	7.82	0.00
Intervention length: ≤4 vs >4 mo	−0.36 (−0.81 to 0.08)	.16	37.77	14.60
Goal set for PA: yes vs no	−0.11 (−0.62 to 0.40)	.66	43.01	0.00
Uptake: ≥80% vs <80%	−0.23 (−0.69 to 0.23)	.32	41.85	0.00
Use of theoretical concept: yes vs no	−0.26 (−0.63 to 0.11)	.16	15.82	0.57
Studies with low risk of bias: yes vs no^b^	0.17 (−0.35 to 0.69)	.49	8.21	4.28
Multivariable				
Type of tracker	0.20 (0.02 to 0.32)	.004	NA	NA
Outcome measure	−0.01 (−0.10 to 0.03)	.06	NA	NA
Consultations with facilitators (delivery)	−0.04 (−0.11 to −0.01)	.01	NA	NA
Sex	0.48 (0.01 to 0.96)	.046	NA	NA
Model fit	χ^2^_3_ = 24.18	.03	NA	NA

Abbreviations: MVPA, moderate-to-vigorous physical activity; NA, not applicable; PA, physical activity.

^a^ *R*^2 ^is the estimated proportion of variance in the dependent variable.

^b^ Low risk of bias was classified as if all risk of bias domains were judged as low risk.

**Figure 2.**
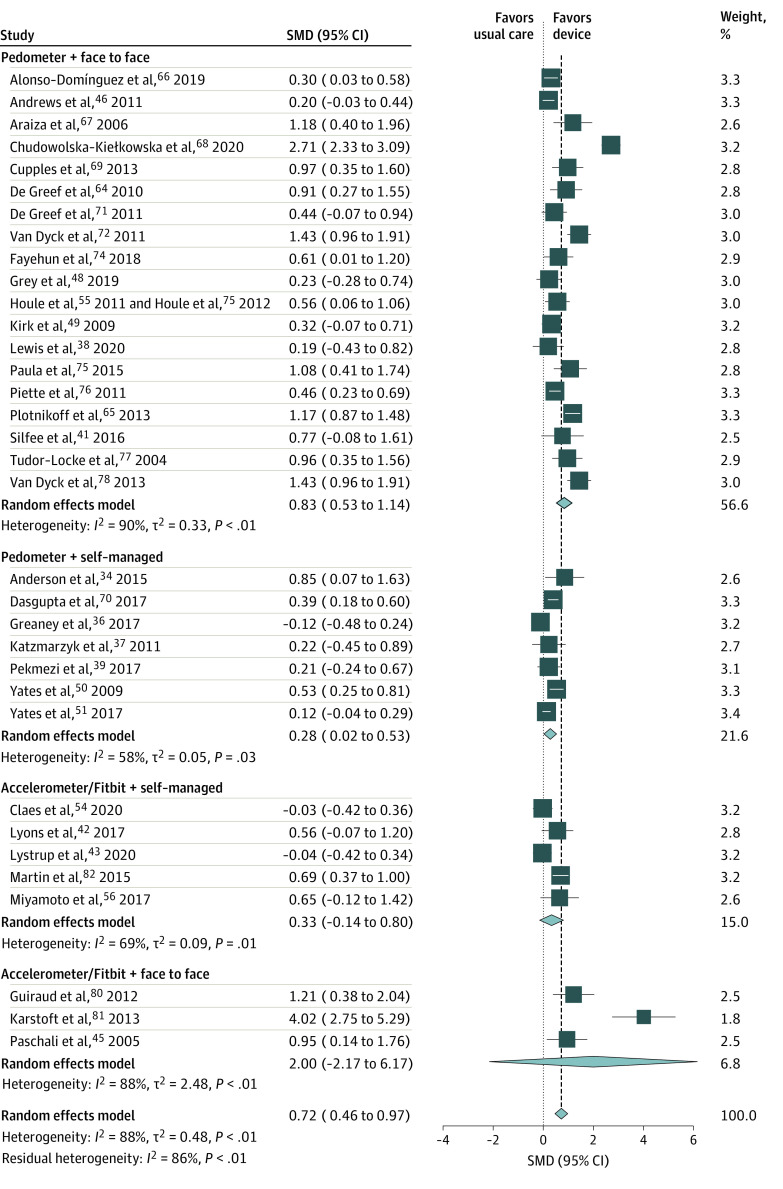
Subgroup Meta-analysis of Delivery and Consultation Type by Intervention Markers indicate standardized mean differences (SMDs), with the size of the markers reflecting weights; horizontal lines indicate 95% CIs; and diamonds indicate pooled means, with the points of the diamonds indicating 95% CIs of the pooled means. The vertical dashed line indicates the point at which there was no difference between intervention and usual care (null effect).

A post hoc subgroup analysis was conducted using the enhanced consultation and monitoring device variables whereby studies were divided into 4 groups to best visualize the results of the metaregression analyses (Figure 2). Pedometer interventions that incorporated consultations with health care professionals were associated with increased PA (SMD, 0.83; 95% CI, 0.53-1.14) as were unsupervised pedometer interventions (SMD, 0.28; 95% CI, 0.02-0.53). Accelerometer and/or fitness tracker interventions with consultations and without consultations were not associated with increased PA.

#### Secondary Outcomes

Interventions with wearable activity trackers were associated with statistically significant reductions in blood glucose levels compared with comparators (mean difference, −0.14%; 95% CI, −0.27% to −0.01%) (Table 2). Pedometer-based interventions had the strongest association with improved blood glucose level (mean difference, −0.19; 95% CI, −0.35 to −0.03); however, accelerometer performance was not associated with improved blood glucose level in 5 studies.^39,40,41,42,43^ There were no associations of wearable activity tracker interventions with systolic blood pressure, diastolic blood pressure, total cholesterol level, high-density lipoprotein cholesterol level, low-density lipoprotein cholesterol level, BMI, and weight.

#### Publication Bias

Publication bias was not obvious for any intervention by visual inspection of the funnel plots (eFigure 4 in the Supplement) and as indicated by the Egger statistic (test for funnel plot asymmetry: pedometers: *z* = 0.97, *P* = .33; accelerometers: *z* = 0.49, *P* = .62). The trim-and-fill method also confirmed no evidence of publication bias. All forest plots are provided in eFigure 5 in the Supplement.

#### Narrative Synthesis

Three pedometer studies^37,44,45^ and 1 accelerometer study^36^ reported nonamenable data for meta-analysis. Two of these studies^36,44^ reported an association with a greater number of steps, and the other 2 studies^37,45^ reported no association with a greater number of steps between intervention and comparator group.

## Discussion

This systematic review and meta-analysis found that interventions using wearable activity trackers were associated with significant improvements in PA among people with cardiometabolic conditions compared with individuals who received usual care. Interventions that combined the use of activity trackers with additional components, such as regular consultation sessions with a health care professional (face to face or remotely), had the strongest associations with PA improvement. The PA improvements were more pronounced among male participants.

Pedometer use had a significant association with improved PA when used in the context of interventions that involved regular consultations with a health care professional compared with self-monitoring interventions without consultations. Accelerometer and fitness tracker interventions were not associated with PA levels compared with comparators when simple self-monitoring or regular consultation-based interventions were used. However, the current comparison involved only 8 studies^35,36,41,57,65,69,73,77^ compared with 26 pedometer studies^35,38,41,43,45,46,48,49,53,55,57,64,65,66,67,68,69,71,72,74,75,76,77,78,79,80,81^ and few patients, and although the effect size was larger for accelerometer and fitness tracker interventions (0.92 vs 0.68 for the pedometer), the 95% CI was wider than that for pedometers. All these factors should be considered when interpreting the efficacy of the interventions. Pedometers are often criticized because they do not measure daily steps precisely enough but may be more suited in the short term for patients with high-risk conditions with an objective of reaching a certain number of steps per day. Moreover, interventions using pedometers typically measured PA using steps per day, whereas interventions using accelerometer or fitness trackers measured PA using MVPA. Thus, the choice of PA measurement (steps per day vs MVPA) might account for the differential performance of these interventions.

Some findings showed that interventions were associated with improved PA levels when they included consultations and longer periods of PA engagement (ie, longer and more regular sessions). However, 9 studies^38,43,44,48,50,69,70,76,81^ did not report the total PA engagement time per session. Interventions were also associated with reduced blood glucose levels. Although the change was statistically significant compared with the usual care group, the clinical relevance of the magnitude of change was small. Other secondary health outcomes, such as blood pressure, cholesterol levels, weight, and BMI, were not associated with the intervention.

The findings of this review are consistent with the results of earlier systematic reviews^17,48^ involving populations at high risk that suggested improvements in PA levels at short-term follow-up assessments when using wearable trackers. One review^48^ reported an association of a wearable tracker intervention with increased PA level (SMD, 0.57; 95% CI, 0.24-0.91) but was based solely on people with type 2 diabetes. Furthermore, a recent systematic review and meta-analysis by Brickwood et al^23^ of consumer-based wearable activity trackers in general populations indicated an association with improved PA levels, but availability of long-term follow-up data was limited. Although that study was similar to the present study with regard to the focus on electronic devices for monitoring PA, the population does not overlap with that in the present study; the study by Brickwood et al^23^ focused on studies conducted among healthy general populations and not among those at risk for chronic conditions. Another study^18^ of wearable devices, mostly including fitness trackers, found that these devices were associated with improved physical health in clinical populations with cardiometabolic diseases. None of the aforementioned reviews used metaregressions to robustly examine factors associated with the effectiveness of these interventions. Findings from the current analysis support the use of interventions that contain consultation sessions with health professionals for boosting the PA benefits for people with cardiometabolic conditions.

In this study, interventions of the use of wearable activity trackers and in particular pedometers were associated with greater PA levels per day among people with cardiometabolic conditions. Nevertheless, the improvements were generally lower than those recommended in the *2018 Physical Activity Guidelines Advisory Committee Scientific Report* by the US Department of Health and Human Services^50^ and in other recommendations from global governments and agencies.^51^ For instance, the National Obesity Forum UK classified 3000 to 6000 steps per day as sedentary, the Northern Irelands Public Health Agency promoted an additional 3000 steps, and the America on the Move program suggested an additional 2000 steps each day to stop weight gain.^77^ For accelerometers and fitness trackers, public health guidelines have endorsed 30 minutes (up to 60 minutes) per day (or 150-210 minutes per week) in MVPA, typically in minimal 10-minute bouts.^52,53^ However, MVPA time engagement could not be assessed because the total session times were reported inconsistently or sometimes not at all reported in studies.

The findings of the present study suggest that interventions that combine the use of monitoring devices (particularly pedometers) with regular consultations with health care professionals predominantly among male participants are associated with the greatest PA improvements and that these interventions may help reach the recommendations of governments and agencies for people with cardiometabolic conditions. Giving feedback and lifestyle advice to patients regularly may support the effectiveness of these interventions. A previous study^54^ suggested positive associations of multimodal pedometer interventions with PA levels in a variety of populations, including those with type 2 diabetes and cardiac conditions, but to our knowledge, this was the first study to assess the association of face-to-face consultations and measurement with PA levels in people with cardiometabolic conditions. These findings warrant consideration in future trials and further investigation using more robust methods, such as meta-analyses of individual participant data. The choice of measurement outcome (steps or MVPA) and their performance differences also need to be better understood in these patient populations.

Only 1 study^55^ reported data on the association of a pedometer-based intervention with PA levels recorded after a 1-year follow-up period. Long-term follow-up assessments are needed to generate evidence regarding the sustainability of the association over time. Providing longer-term assessments of these interventions may have greater potential to impact the clinical outcome performance and might provide more information on the intervention program than current short-term assessments.

### Strengths and Limitations

This study has strengths. First, 4 major databases were searched for relevant literature. Second, the analysis used well-established statistical methods, including multilevel, multivariable metaregression, to explore the association of certain moderators with the outcome of interest.

This study also has limitations. Because the included studies did not provide amenable data for scores, correlations, or SD changes before and after the intervention, the effects of changes at baseline could not be considered in the meta-analysis, which could have affected the results. Furthermore, because the PA promotion technique was poorly reported across studies, we were not able to assess this in regression analysis. Metaregressions were performed to explore the heterogeneity observed in the main analyses, but important uncertainties remained regarding risk of bias assessments with many unclear domains, and participant characteristics, such as age and sex, were based only on aggregate data. In addition, it was not possible to assess for commercial bias because few studies declared their commercial interests. Because of the heterogeneous nature of cardiovascular disease index among the 8 trials^35,36,41,57,65,69,73,77^ and the limited number of trials that focused on participants with overweight or obesity, the trials were combined under the definition of a cardiometabolic condition. Only study populations at risk for a cardiometabolic condition were accepted as part of our inclusion criteria; however, this is a potential limitation because studies that did not explicitly report this may have been missed. In addition, it was not possible to look at behavior change outcomes, such as those reported in line with the theoretical domains framework, because only 1 trial^49^ mentioned explicitly in their aims that such outcomes would be collected.

## Conclusions

In this systematic review and meta-analysis of individuals with cardiometabolic conditions, interventions that combined activity trackers (especially pedometers) with complementary intervention components, such as consultations with health care professionals, were significantly associated with increased levels of PA. Understanding how to improve these interventions further for greater PA improvements in the longer term may have implications in the care of people with cardiometabolic conditions.

## Notes

Supplement. **eFigure 1.** Forest Plot of Accelerometer/Fitbit vs Pedometer-Based Interventions With Prediction Interval **eFigure 2.** Forest Plot of Pedometer-Based Interventions on Mean Difference Scale **eFigure 3.** Cumulative Forest Plot of PA Performance Based on Total PA Engagement Time (Combined by Total Minutes) **eFigure 4.** Individual Funnel Plots of Accelerometer/Fitbit and Pedometer-Based Interventions **eFigure 5.** All Forest Plots **eTable 1.** Search Strategies **eTable 2.** Citations of Eligible Studies for Review **eTable 3.** Summary of Intervention Characteristics by Study **eTable 4.** Risk of Bias Study-by-Study Summary and Overall, by Each Domain
