# Supplementary material for: Interventions Using Wearable Physical Activity Trackers Among Adults With Cardiometabolic Conditions: A Systematic Review and Meta-analysis
Source: JAMA Netw Open. 2021 Jul 20;4(7):e2116382. doi: 10.1001/jamanetworkopen.2021.16382 (PMC9387744; doi:10.1001/jamanetworkopen.2021.16382)
Supplement: Supplement. — eFigure 1. Forest Plot of Accelerometer/Fitbit vs Pedometer-Based Interventions With Prediction Interval eFigure 2. Forest Plot of Pedometer-Based Interventions on Mean Difference Scale eFigure 3. Cumulative Forest Plot of PA Performance Based on Total PA Engagement Time (Combined by Total Minutes) eFigure 4. Individual Funnel Plots of Accelerometer/Fitbit and Pedometer-Based Interventions eFigure 5. All Forest Plots eTable 1. Search Strategies eTable 2. Citations of Eligible Studies for Review eTable 3. Summary of Intervention Characteristics by Study eTable 4. Risk of Bias Study-by-Study Summary and Overall, by Each Domain [file jamanetwopen-e2116382-s001.pdf]

Supplementary Online Content

Hodkinson A, Kontopantelis E, Adeniji C, et al. Interventions using wearable physical activity trackers among adults with cardiometabolic conditions: a systematic review and meta-analysis. *JAMA Netw Open*. 2021;4(7):e2116382. doi:10.1001/jamanetworkopen.2021.16382

eFigure 1. Forest Plot of Accelerometer/Fitbit vs Pedometer-Based Interventions With Prediction Interval

eFigure 2. Forest Plot of Pedometer-Based Interventions on Mean Difference Scale

eFigure 3. Cumulative Forest Plot of PA Performance Based on Total PA Engagement Time (Combined by Total Minutes)

eFigure 4. Individual Funnel Plots of Accelerometer/Fitbit and Pedometer-Based Interventions

eFigure 5. All Forest Plots

eTable 1. Search Strategies

eTable 2. Citations of Eligible Studies for Review

eTable 3. Summary of Intervention Characteristics by Study

eTable 4. Risk of Bias Study-by-Study Summary and Overall, by Each Domain

This supplementary material has been provided by the authors to give readers additional information about their work.

eFigure 1: Forest plot of Accelerometer/Fitbit vs pedometer-based interventions with prediction interval

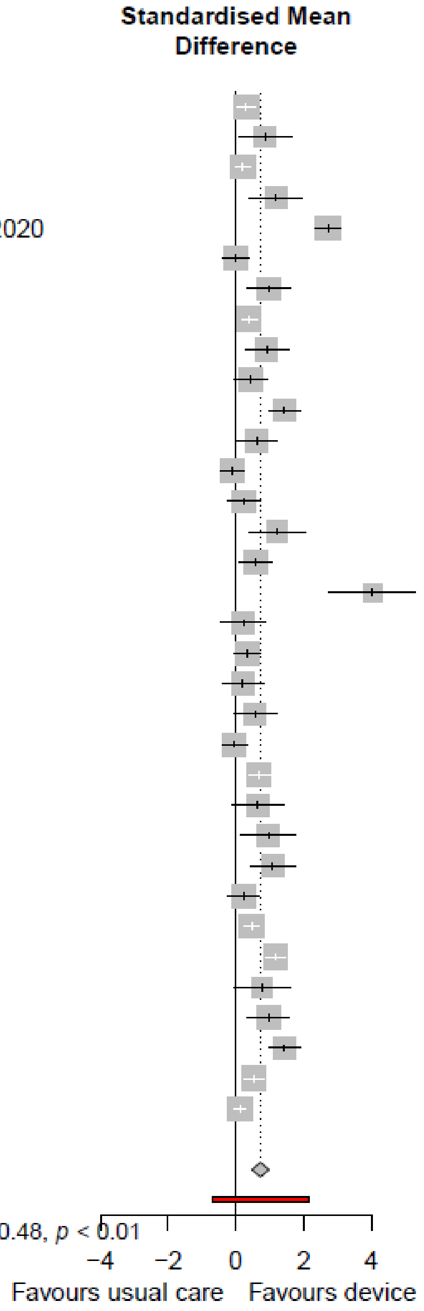

Study	SMD	95%−CI	Weight
Alonso−Dominguez 2019	0.30	[0.03; 0.58]	3.3%
Anderson 2015	0.85	[0.07; 1.63]	2.6%
Andrews 2011	0.20	[−0.03; 0.44]	3.3%
Araiza 2006	1.18	[0.40; 1.96]	2.6%
Chudowolska−Kielkowska 2020	2.71	[2.33; 3.09]	3.2%
Claes 2020	−0.03	[−0.42; 0.36]	3.2%
Cupples 2013	0.97	[0.35; 1.60]	2.8%
Dasgupta 2017	0.39	[0.18; 0.60]	3.3%
De Greef 2010	0.91	[0.27; 1.55]	2.8%
De Greef 2011 (1)	0.44	[−0.07; 0.94]	3.0%
De Greef 2011 (2)	1.43	[0.96; 1.91]	3.0%
Fayehun 2018	0.61	[0.01; 1.20]	2.9%
Greaney 2017	−0.12	[−0.48; 0.24]	3.2%
Grey 2019	0.23	[−0.28; 0.74]	3.0%
Guiraud 2012	1.21	[0.38; 2.04]	2.5%
Houle 2011, 2012	0.56	[0.06; 1.06]	3.0%
Karstoft 2013	4.02	[2.75; 5.29]	1.8%
Katzmarzyk 2011	0.22	[−0.45; 0.89]	2.7%
Kirk 2009	0.32	[−0.07; 0.71]	3.2%
Lewis 2020	0.19	[−0.43; 0.82]	2.8%
Lyons 2017	0.56	[−0.07; 1.20]	2.8%
Lystrup 2020	−0.04	[−0.42; 0.34]	3.2%
Martin 2015	0.69	[0.37; 1.00]	3.2%
Miyamoto 2017	0.65	[−0.12; 1.42]	2.6%
Paschali 2005	0.95	[0.14; 1.76]	2.5%
Paula 2015	1.08	[0.41; 1.74]	2.8%
Pekmezi 2017	0.21	[−0.24; 0.67]	3.1%
Piette 2011	0.46	[0.23; 0.69]	3.3%
Plotnikoff 2013	1.17	[0.87; 1.48]	3.3%
Silfee 2016	0.77	[−0.08; 1.61]	2.5%
Tudor−Locke 2004	0.96	[0.35; 1.56]	2.9%
Van Dyck 2013	1.43	[0.96; 1.91]	3.0%
Yates 2009	0.53	[0.25; 0.81]	3.3%
Yates 2017	0.12	[−0.04; 0.29]	3.4%
**Random effects model**	**0.72**	**[0.46; 0.97]**	**100.0%**
**Prediction interval**		**[−0.72; 2.16]**	

Heterogeneity: I^2 = 88%, τ^2 = 0.48, p < 0.01

© 2021 Hodkinson A et al. *JAMA Network Open.*

eFigure 2: Forest plot of pedometer-based interventions on mean difference scale

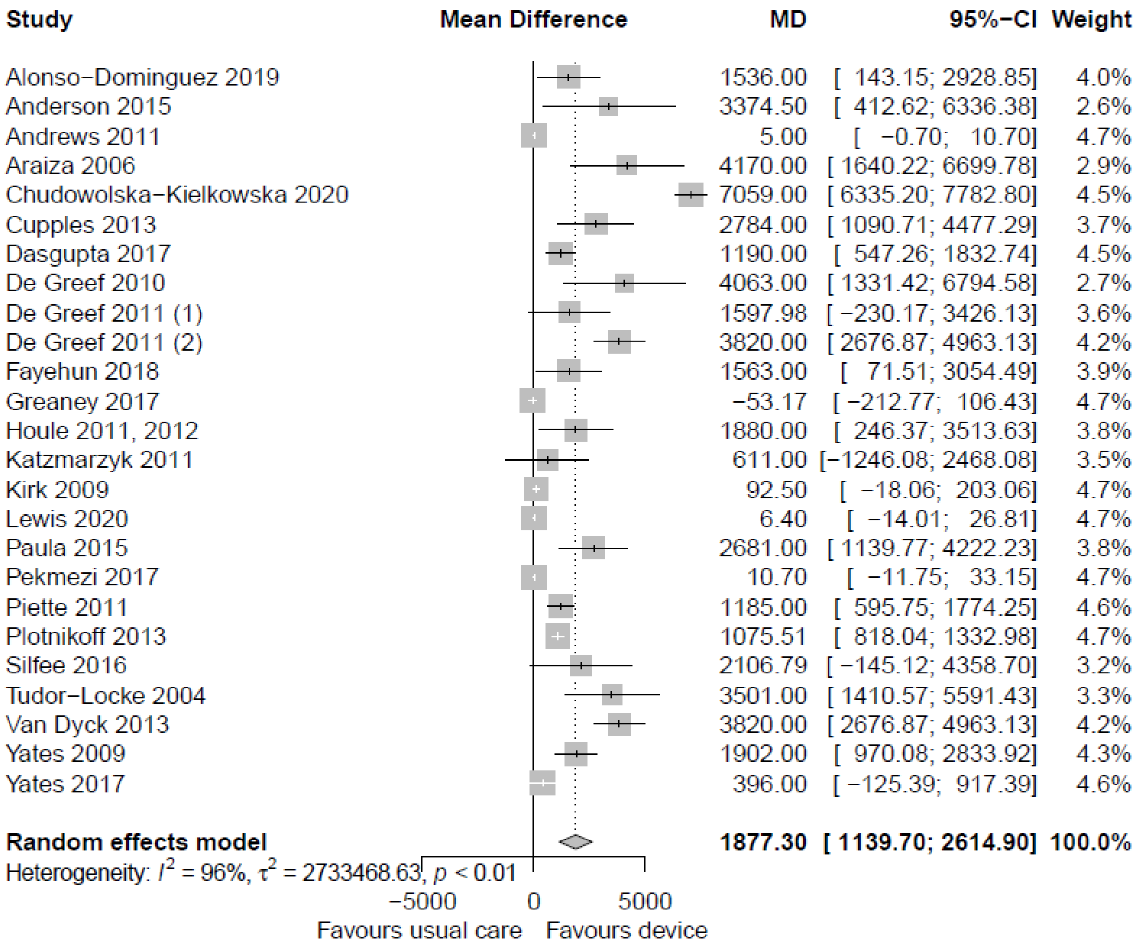

© 2021 Hodkinson A et al. *JAMA Network Open.*

eFigure 3: Cumulative forest plot ordered by highest-to-lowest total session time engagement in PA (recorded by total minutes)

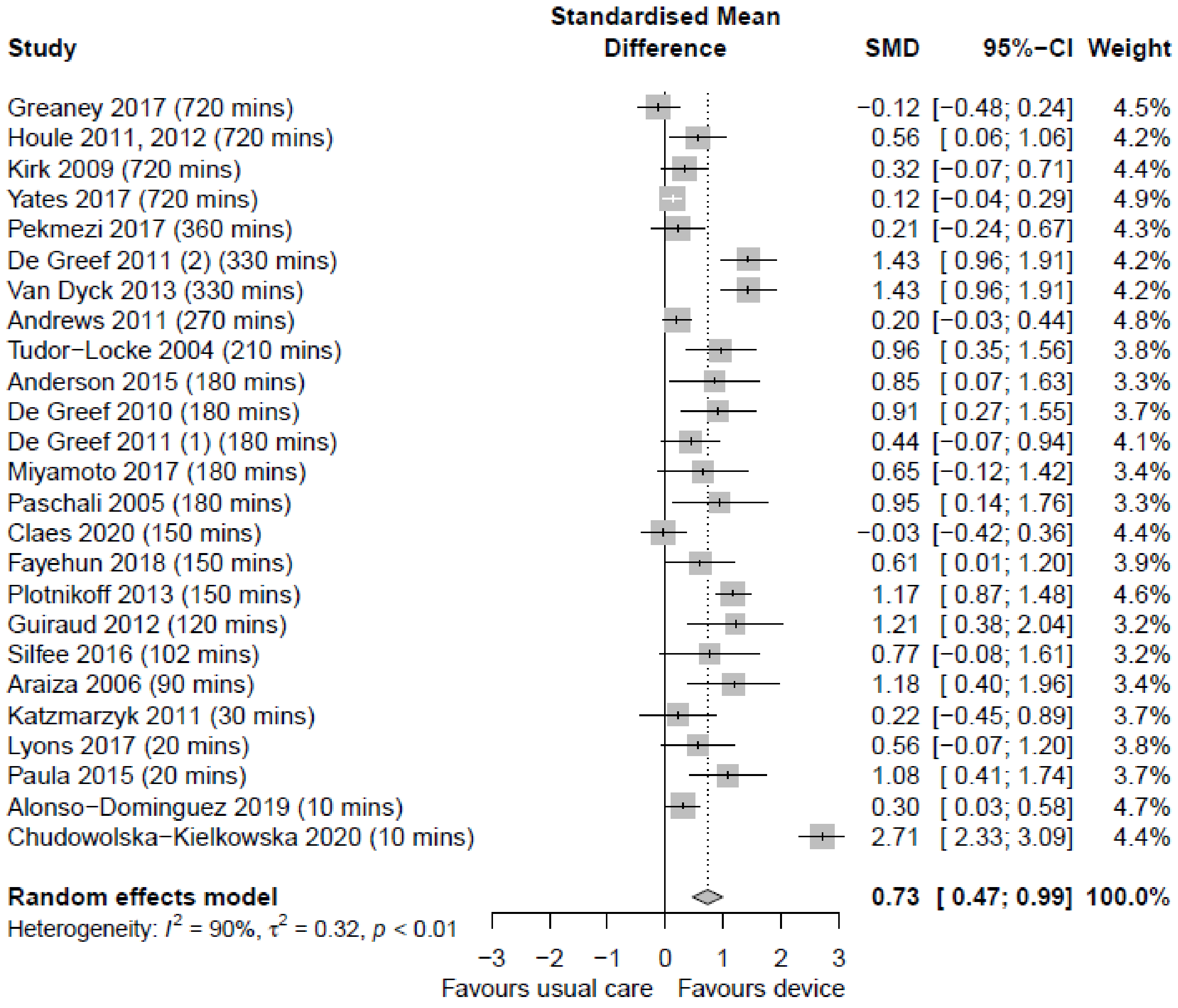

© 2021 Hodkinson A et al. *JAMA Network Open.*

eFigure 4: Individual funnel plots of accelerometer/Fitbit and pedometer-based interventions

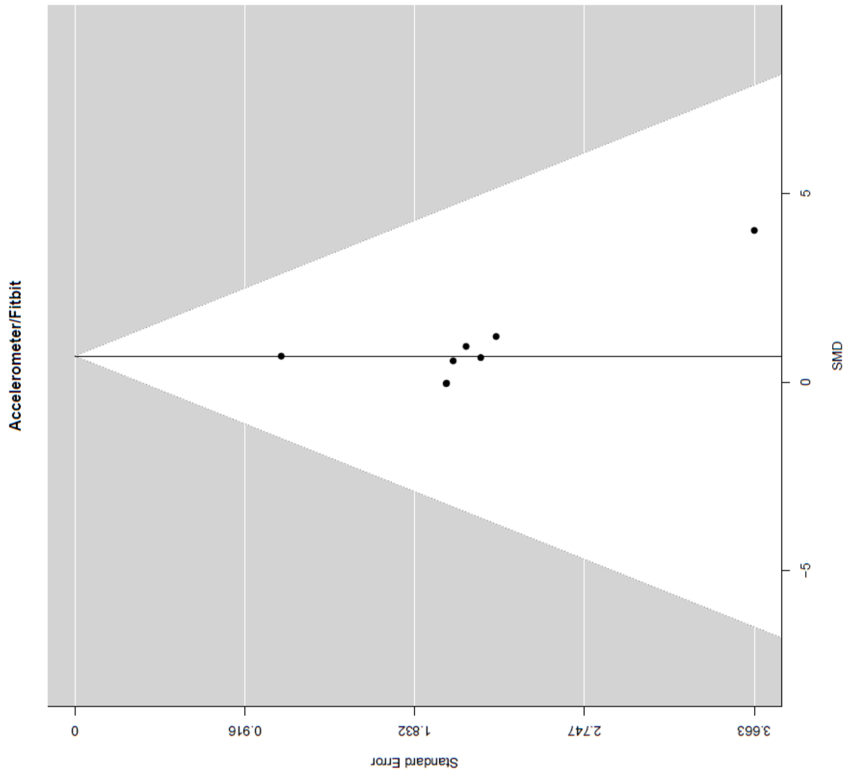

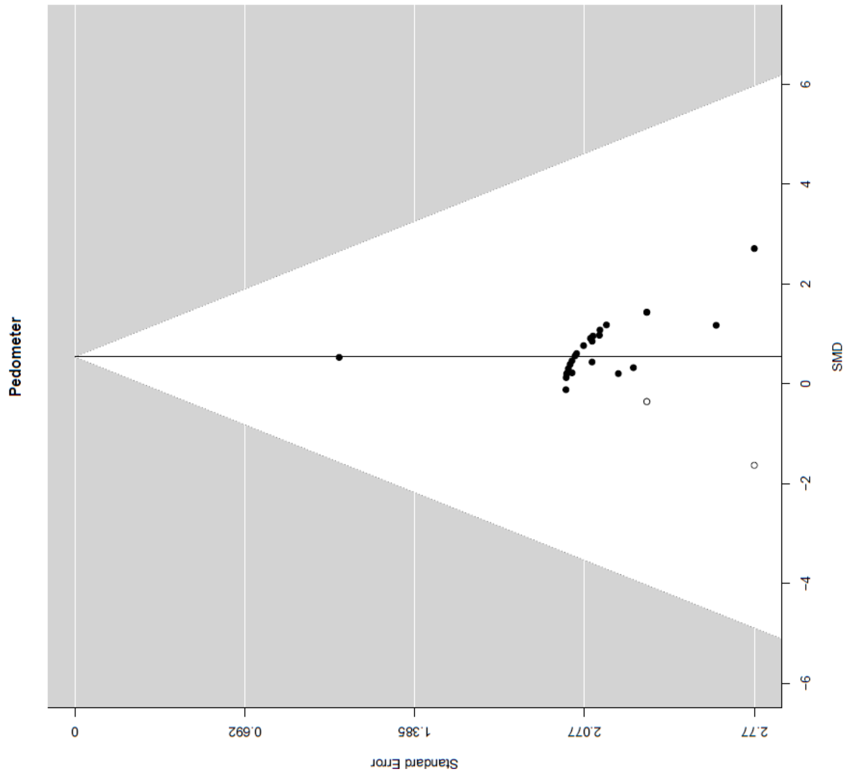

© 2021 Hodkinson A et al. *JAMA Network Open.*

Meta regression results (egger's and trim & fill)

Pedometers:

```
Random/mixed effects version of the Eggar test:

Regression Test for Funnel Plot Asymmetry

model:     mixed-effects meta-regression model
predictor: standard error

test for funnel plot asymmetry: z = 0.9660, p = 0.3340


Trim and fill:

Estimated number of missing studies on the left side: 0 (SE = 2.9654)

Random-Effects Model (k = 24; tau^2 estimator: REML)

tau^2 (estimated amount of total heterogeneity): 0 (SE = 1.3174)
tau (square root of estimated tau^2 value):      0
I^2 (total heterogeneity / total variability):   0.00%
H^2 (total variability / sampling variability):  1.00

Test for Heterogeneity:
Q(df = 23) = 1.4798, p-val = 1.0000

Model Results:

estimate      se    zval    pval    ci.lb   ci.ub
  0.6179  0.4335  1.4254  0.1541  -0.2318  1.4675

---
Signif. codes:  0 '***' 0.001 '**' 0.01 '*' 0.05 '.' 0.1 ' ' 1
```

Accelerometers/Fitbits:

```
Random/mixed effects version of the Eggar test:

Regression Test for Funnel Plot Asymmetry

model:     mixed-effects meta-regression model
predictor: standard error

test for funnel plot asymmetry: z = 0.4884, p = 0.6253

Trim and fill:

Estimated number of missing studies on the left side: 0 (SE = 1.9044)

Random-Effects Model (k = 8; tau^2 estimator: REML)

tau^2 (estimated amount of total heterogeneity): 0 (SE = 1.7815)
tau (square root of estimated tau^2 value):      0
I^2 (total heterogeneity / total variability):   0.00%
H^2 (total variability / sampling variability):  1.00

Test for Heterogeneity:
Q(df = 7) = 1.2054, p-val = 0.9908

Model Results:
```

© 2021 Hodkinson A et al. *JAMA Network Open.*

```
estimate      se    zval    pval    ci.lb   ci.ub
  0.6552  0.6655  0.9845  0.3249  -0.6492  1.9596

---
Signif. codes:  0 ‘***’ 0.001 ‘**’ 0.01 ‘*’ 0.05 ‘.’ 0.1 ‘ ’ 1
```

© 2021 Hodkinson A et al. *JAMA Network Open.*

eFigure 5: All forest plots

Physical activity measurement used for primary outcome (Steps vs. MVPA)

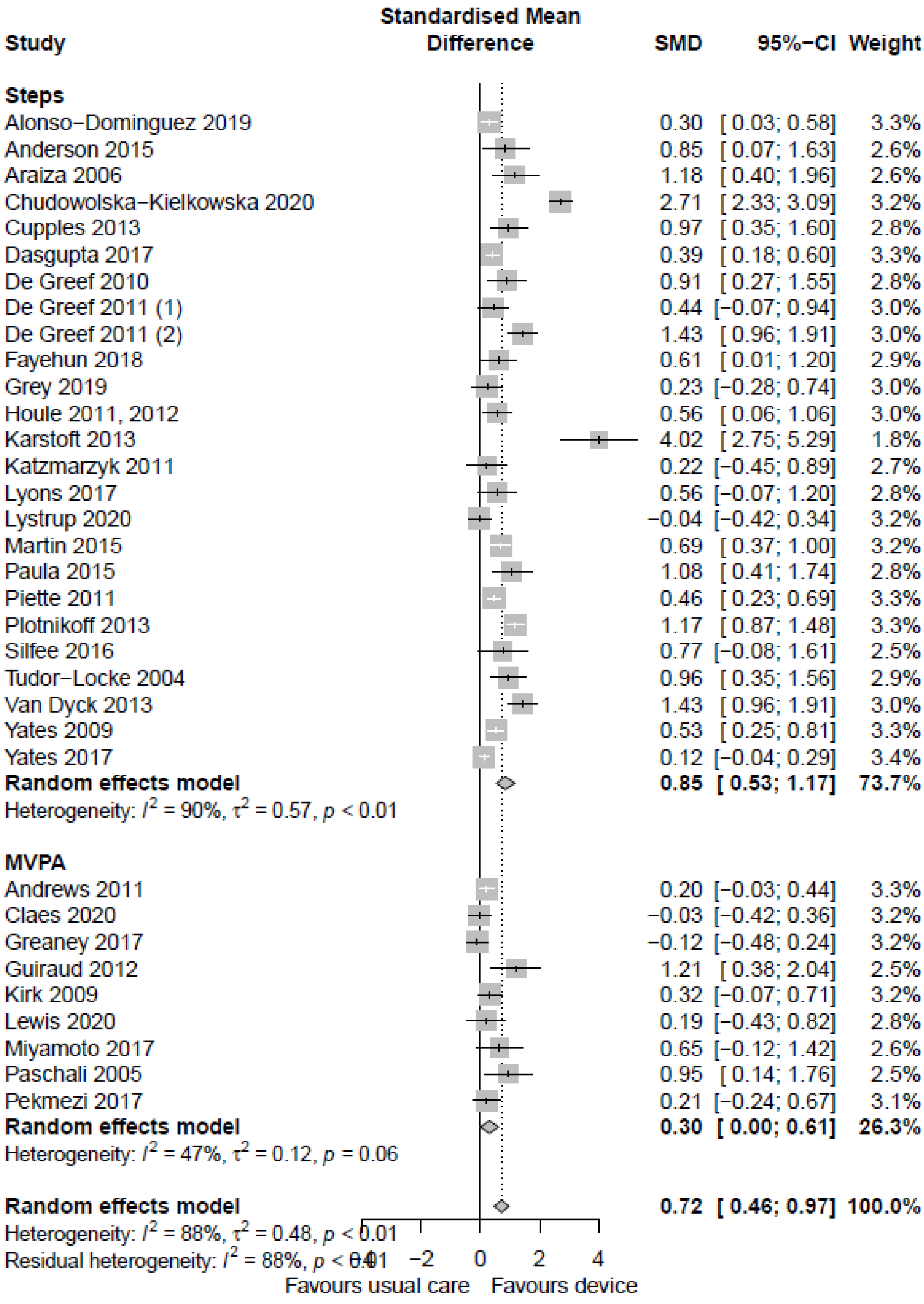

© 2021 Hodkinson A et al. *JAMA Network Open.*

Physical activity by delivery type (FTF consultation vs. self-managed)

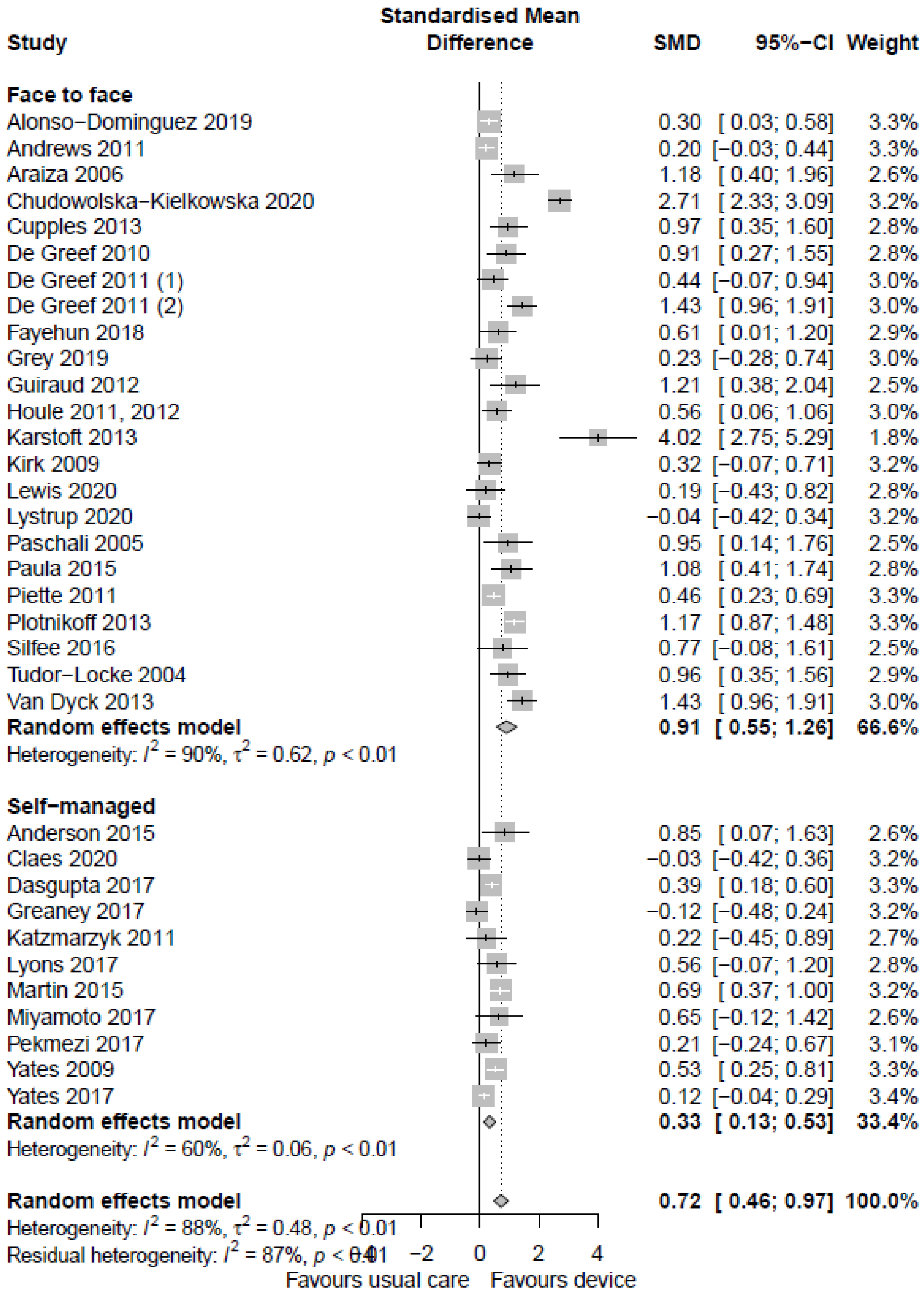

© 2021 Hodkinson A et al. *JAMA Network Open.*

Blood (sugar) glucose (hba1c %)

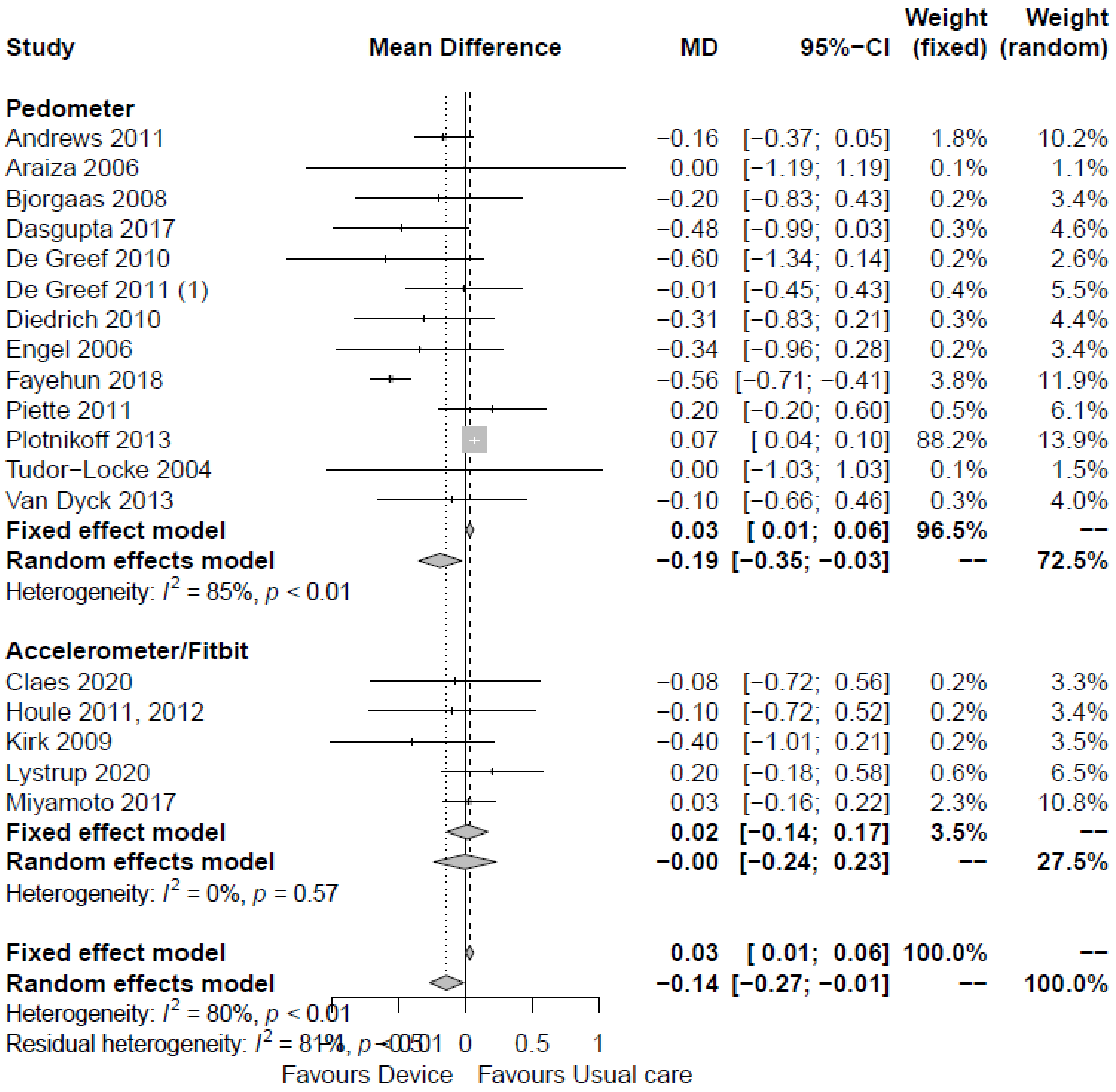

© 2021 Hodkinson A et al. *JAMA Network Open.*

Blood pressure (SBP and DBP)

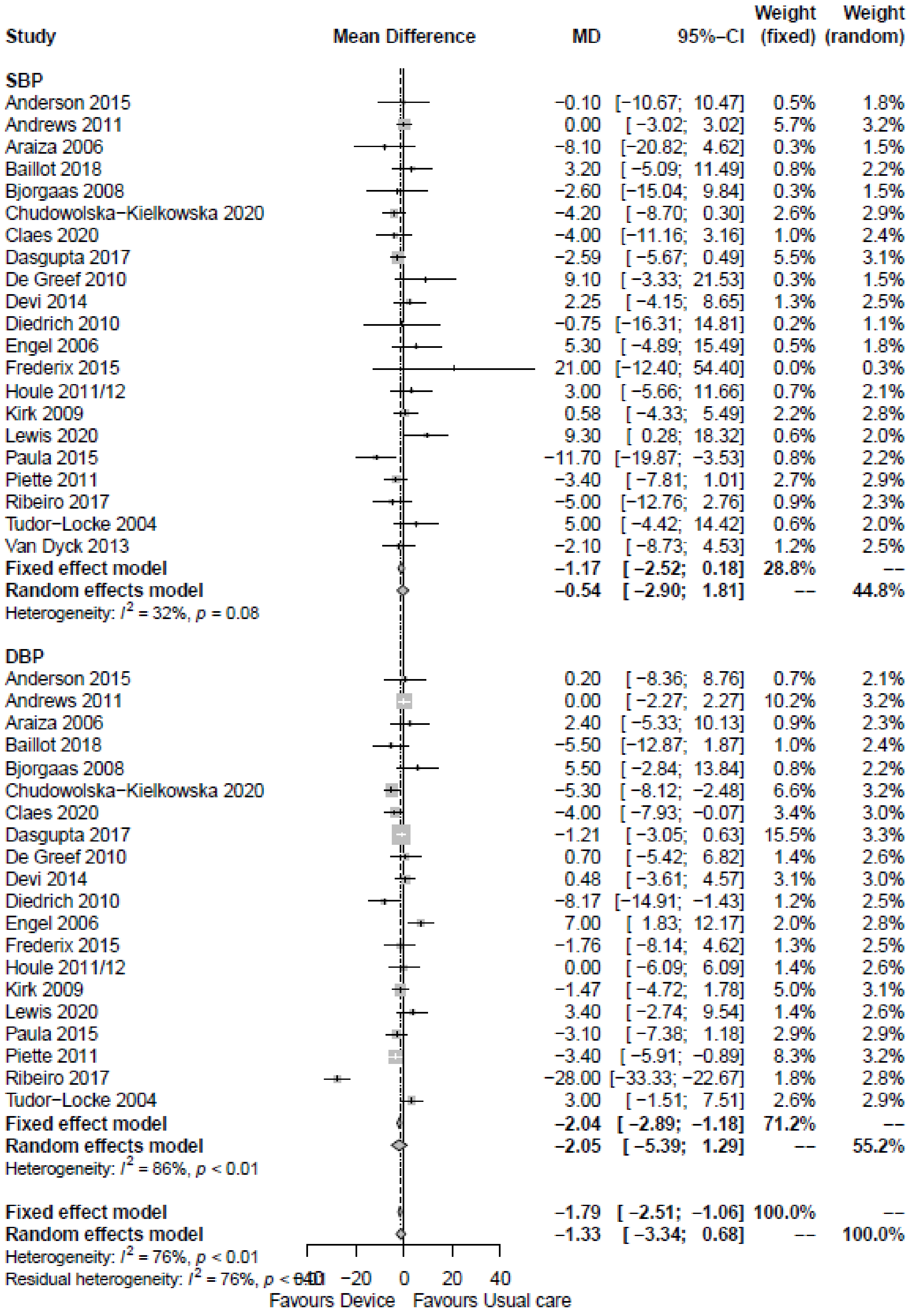

© 2021 Hodkinson A et al. *JAMA Network Open.*

Cholesterol (total, HDL, LDL)

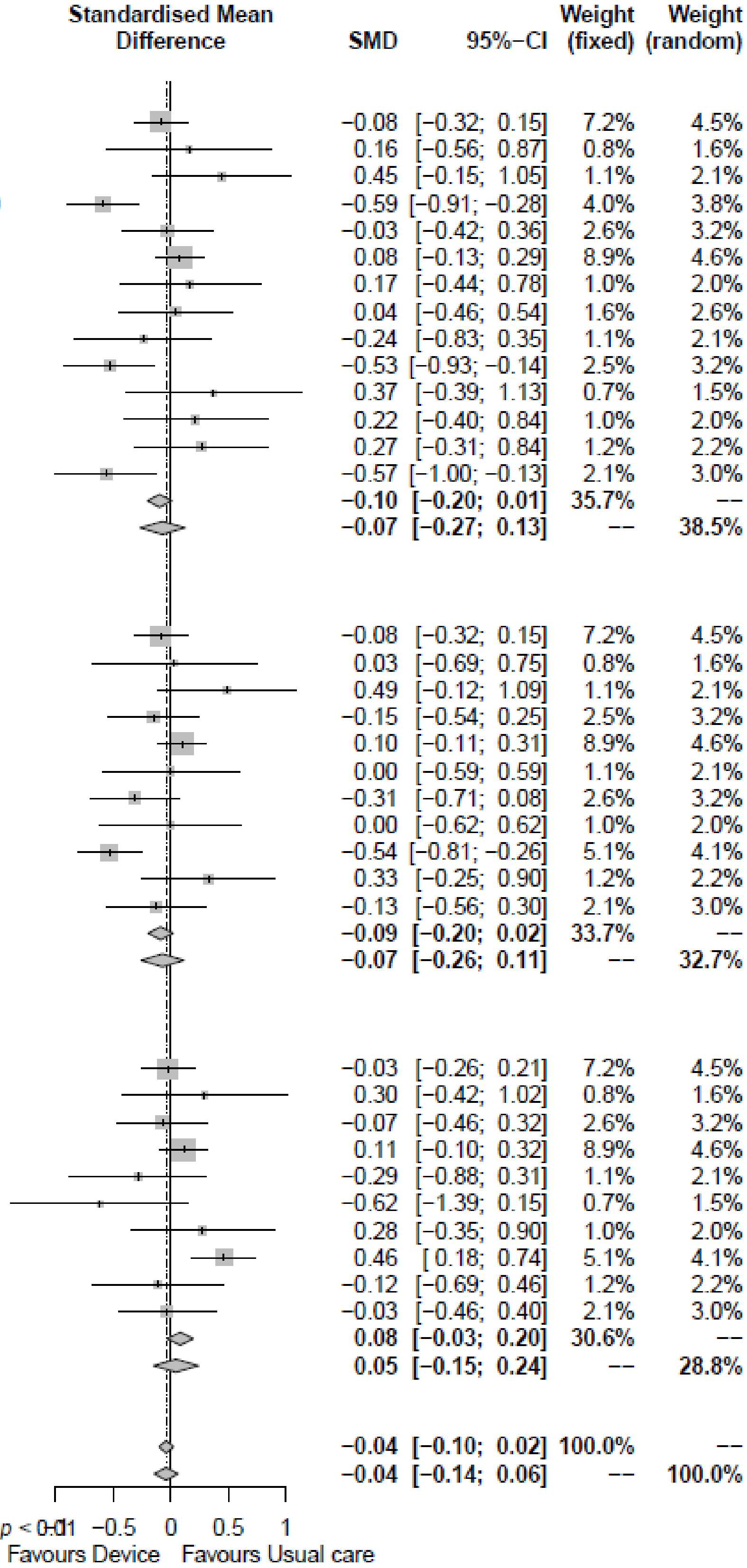

Study	SMD	95%−CI	Weight (fixed)	Weight (random)
**Total Cholesterol**				
Andrews 2011	−0.08	[−0.32; 0.15]	7.2%	4.5%
Araiza 2006	0.16	[−0.56; 0.87]	0.8%	1.6%
Bjorgaas 2008	0.45	[−0.15; 1.05]	1.1%	2.1%
Chudowolska−Kielkowska 2020	−0.59	[−0.91; −0.28]	4.0%	3.8%
Claes 2020	−0.03	[−0.42; 0.36]	2.6%	3.2%
Dasgupta 2017	0.08	[−0.13; 0.29]	8.9%	4.6%
De Greef 2010	0.17	[−0.44; 0.78]	1.0%	2.0%
De Greef 2011(1)	0.04	[−0.46; 0.54]	1.6%	2.6%
Houle 2011/12	−0.24	[−0.83; 0.35]	1.1%	2.1%
Kirk 2009	−0.53	[−0.93; −0.14]	2.5%	3.2%
Miyamoto 2017	0.37	[−0.39; 1.13]	0.7%	1.5%
Paula 2015	0.22	[−0.40; 0.84]	1.0%	2.0%
Tudor−Locke 2004	0.27	[−0.31; 0.84]	1.2%	2.2%
Van Dyck 2013	−0.57	[−1.00; −0.13]	2.1%	3.0%
**Fixed effect model**	**−0.10**	**[−0.20; 0.01]**	**35.7%**	**--**
**Random effects model**	**−0.07**	**[−0.27; 0.13]**	**--**	**38.5%**
Heterogeneity: $I^2 = 57\%$, $p < 0.01$				
**HDL (mg/dl)**				
Andrews 2011	−0.08	[−0.32; 0.15]	7.2%	4.5%
Araiza 2006	0.03	[−0.69; 0.75]	0.8%	1.6%
Bjorgaas 2008	0.49	[−0.12; 1.09]	1.1%	2.1%
Claes 2020	−0.15	[−0.54; 0.25]	2.5%	3.2%
Dasgupta 2017	0.10	[−0.11; 0.31]	8.9%	4.6%
Houle 2011/12	0.00	[−0.59; 0.59]	1.1%	2.1%
Kirk 2009	−0.31	[−0.71; 0.08]	2.6%	3.2%
Paula 2015	0.00	[−0.62; 0.62]	1.0%	2.0%
Plotnikoff 2013	−0.54	[−0.81; −0.26]	5.1%	4.1%
Tudor−Locke 2004	0.33	[−0.25; 0.90]	1.2%	2.2%
Van Dyck 2013	−0.13	[−0.56; 0.30]	2.1%	3.0%
**Fixed effect model**	**−0.09**	**[−0.20; 0.02]**	**33.7%**	**--**
**Random effects model**	**−0.07**	**[−0.26; 0.11]**	**--**	**32.7%**
Heterogeneity: $I^2 = 51\%$, $p = 0.03$				
**LDL (mg/dl)**				
Andrews 2011	−0.03	[−0.26; 0.21]	7.2%	4.5%
Araiza 2006	0.30	[−0.42; 1.02]	0.8%	1.6%
Claes 2020	−0.07	[−0.46; 0.32]	2.6%	3.2%
Dasgupta 2017	0.11	[−0.10; 0.32]	8.9%	4.6%
Houle 2011/12	−0.29	[−0.88; 0.31]	1.1%	2.1%
Miyamoto 2017	−0.62	[−1.39; 0.15]	0.7%	1.5%
Paula 2015	0.28	[−0.35; 0.90]	1.0%	2.0%
Plotnikoff 2013	0.46	[0.18; 0.74]	5.1%	4.1%
Tudor−Locke 2004	−0.12	[−0.69; 0.46]	1.2%	2.2%
Van Dyck 2013	−0.03	[−0.46; 0.40]	2.1%	3.0%
**Fixed effect model**	**0.08**	**[−0.03; 0.20]**	**30.6%**	**--**
**Random effects model**	**0.05**	**[−0.15; 0.24]**	**--**	**28.8%**
Heterogeneity: $I^2 = 39\%$, $p = 0.10$				
**Fixed effect model**	**−0.04**	**[−0.10; 0.02]**	**100.0%**	**--**
**Random effects model**	**−0.04**	**[−0.14; 0.06]**	**--**	**100.0%**
Heterogeneity: $I^2 = 53\%$, $p < 0.01$				
Residual heterogeneity: $I^2 = 51\%$, $p < 0.01$				

© 2021 Hodkinson A et al. *JAMA Network Open.*

BMI

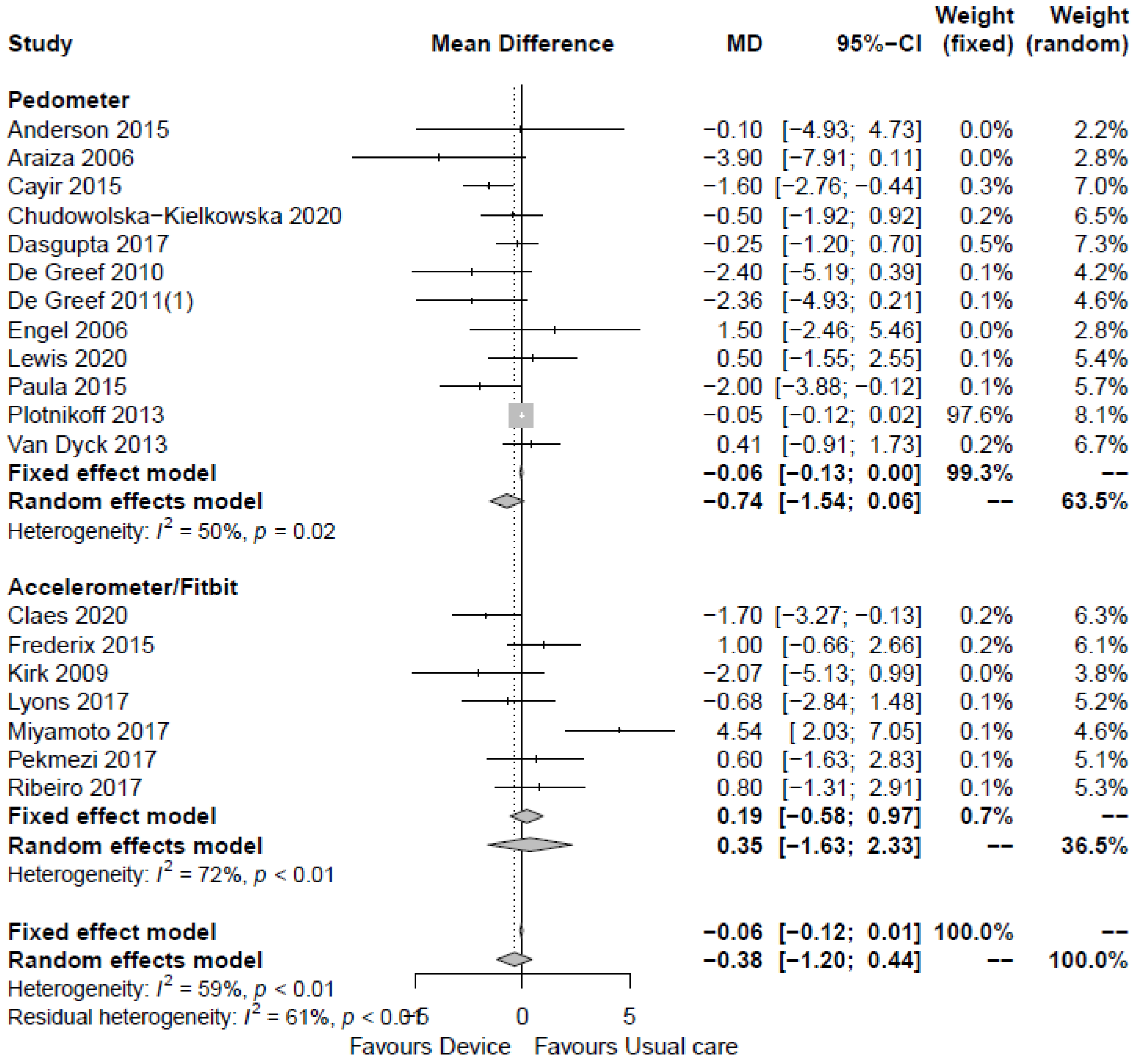

© 2021 Hodkinson A et al. *JAMA Network Open.*

Weight (kg)

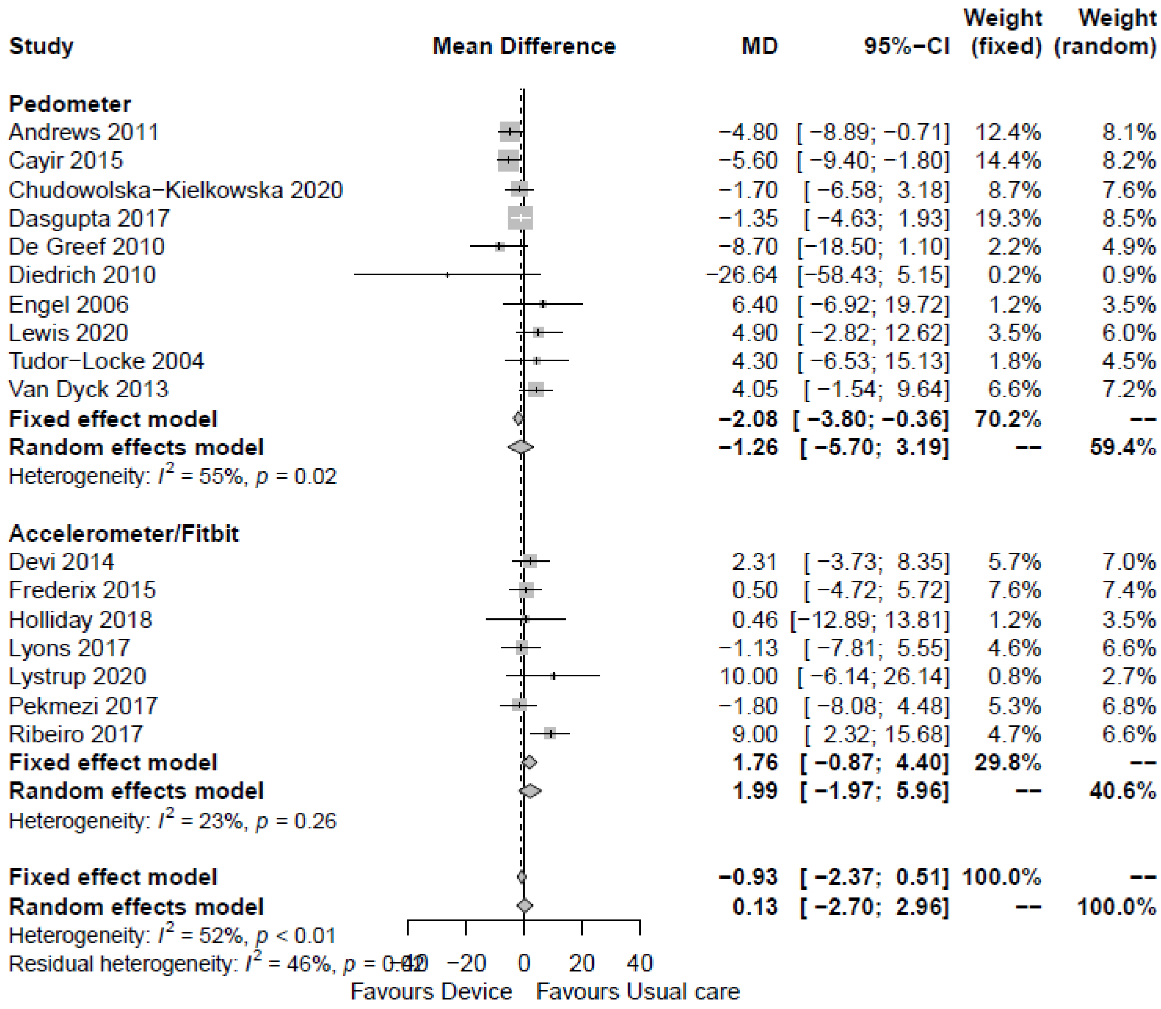

© 2021 Hodkinson A et al. *JAMA Network Open.*

eTable 1: Search strategies

Medline

# ▲	Searches	Results
1	Cardiovascular Diseases/	152632
2	(cardiovascular adj1 disease$).tw.	150229
3	cardiovascular risk factor$.tw.	31197
4	exp heart diseases/	1146729
5	exp Coronary Artery Bypass/	53473
6	exp Myocardial Revascularization/	92869
7	exp heart transplantation/	36070
8	Percutaneous Coronary Intervention/ or Angioplasty, Balloon, Coronary/	54555
9	Heart Valve Prosthesis/	36394
10	Pulmonary embolism/	39358
11	((myocardial or cardiac or heart) adj2 (infarct* or isch?emi*)).tw.	239817
12	(coronary adj2 (syndrome* or disease* or event* or occlusion* or stenos* or thrombo*)).tw.	178348
13	(myocard* adj2 revasculari?ation).tw.	5352
14	(STEMI or NSTEMI).tw.	10192
15	(ST adj2 (elevat* or depress*)).tw.	29827
16	"heart transplant*".tw.	21509
17	angina.tw.	49402
18	(heart adj2 (failure or attack or bypass or disease*)).tw.	294547
19	((heart or cardiac or myocard*) adj2 (fail* or insufficien* or decomp*)).tw.	168633
20	(HFpEF or HFrEF or left ventricular ejection fraction or ((preserved or reduced) adj ejection fraction)).tw.	28582
21	(LV dysfunction or (diastolic adj (dysfunction* or failure*)) or (systolic adj (dysfunction* or failure*))).tw.	17690
22	pulmonary embolism*.tw.	29722
23	CABG.tw.	16083
24	(coronary adj2 bypass).tw.	43973
25	PTCA.tw.	6211
26	angioplast*.tw.	40523
27	PCI.tw.	22324
28	(Percutaneous adj2 intervention*).tw.	32511
29	(stent* adj3 (heart or cardiac*)).tw.	805
30	(heart valve adj1 (device* or artificial or prosthesis)).tw.	667
31	cardiomyopath*.tw.	63613
32	cardiovascular disease*.tw.	149898
33	or/1-32	1570796
34	Diabetes mellitus/	119569
35	diabet*.ti.	306993
36	exp Diabetes Mellitus, Type 2/	137087
37	((type 2 or type ii) adj2 diabet*).ti,ab.	124078
38	((non insulin* depend* or non insulin* depend* or non-insulin?depend* or non insulin?depend*) adj1 diabet*).ti,ab.	9807
39	(T2DM or T2D or TIIDM or TIID or NIDDM or MODY or MODM or AODM).ti,ab.	31996
40	((obes* or overweight) adj5 diabet*).ti,ab.	39051

41	prediabetic state/	7067
42	(prediabetes or pre diabetes or raised glucose intolerance or impaired glucose level$ or impaired glucose tolerance or IGT or impaired fasting glucose or IFT or FPG or fasting plasma glucose or impaired glucose regulation or impaired glucose metabolism or raised glycated haemoglobin or raised glycated hemoglobin or high glycated Hb or hyperglycaemia or hyperglycemia).tw.	75450
43	((prevent* or avoid* or delay* or decreas* or reduc*) adj2 (type II diabetes or type 2 diabetes or T2D or DM or diabetes)).ti,ab.	12238
44	or/34-43	463396
45	exp Obesity/	218805
46	Obese.tw.	112489
47	exp Overweight/	225397
48	(BMI or body mass index).af.	255484
49	Weight gain/	31929
50	(Overweight or over weight or obesity or adipose).af.	416529
51	exp Obesity/pc	19636
52	(body mass index or BMI).mp.	254344
53	or/45-52	595592
54	Randomized Controlled Trial/	520611
55	Clinical Trial/	526309
56	randomized controlled trial.pt. or randomised controlled trial.mp. [mp=title, abstract, original title, name of substance word, subject heading word, floating sub-heading word, keyword heading word, organism supplementary concept word, protocol supplementary concept word, rare disease supplementary concept word, unique identifier, synonyms]	525725
57	controlled clinical trial.pt.	93998
58	trial*.ti,ab.	916198
59	or/54-58	1474132
60	pedomet*.mp.	2396
61	((step* or walk*) adj2 (count* or sensor or meter)).ti,ab.	2850
62	Accelerometry/ or (accelerom* or actimeter or actigraph or actiwatch or GT3X or fitbit).ti,ab.	16461
63	((activit* or move* or motion or energy or exercise) adj2 (monitor* or sens* or detect* or count*)).tw.	63488
64	or/60-63	81215
65	33 or 44 or 53	2390948
66	59 and 64 and 65	2120
**67**	**limit 66 to yr="2000 - current"**	**1926**

© 2021 Hodkinson A et al. *JAMA Network Open.*

Embase

# ▲	Searches	Results
1	Diabetes mellitus/	548816
2	diabet*.ti.	459899
3	exp Diabetes Mellitus, Type 2/	267837
4	((type 2 or type ii) adj2 diabet*).ti,ab.	235422
5	((non insulin* depend* or non insulin* depend* or non-insulin?depend* or non insulin?depend*) adj1 diabet*).ti,ab.	11649
6	(T2DM or T2D or TIIDM or TIID or NIDDM or MODY or MODM or AODM).ti,ab.	70722
7	((obes* or overweight) adj5 diabet*).ti,ab.	70600
8	prediabetic state/	14073
9	(prediabetes or pre diabetes or pre-dm or subclinical diabetic or raised glucose intolerance or impaired glucose level$ or impaired glucose tolerance or IGT or impaired fasting glucose or IFT or FPG or fasting plasma glucose or impaired glucose regulation or impaired glucose metabolism or raised glycated haemoglobin or raised glycated hemoglobin or high glycated Hb or hyperglycaemia or hyperglycemia or without diabet* or without diagnosed diabet*).tw.	145300
10	((prevent* or avoid* or delay* or decreas* or reduc*) adj2 (type II diabetes or type 2 diabetes or T2D or DM or diabetes)).ti,ab.	20813
11	or/1-10	1008275
12	exp Obesity/	532605
13	Obese.tw.	201114
14	(overweight or obese or over-weight or over weight or overeating or over eating or over-eating).ti.	75901
15	exp Overweight/	532605
16	(BMI or body mass index).af.	474490
17	exp weight reduction programs/	2431
18	Weight gain/	87925
19	(Overweight or over weight or obesity or adipose).af.	683883
20	exp Obesity/pc	16147
21	(body mass index or BMI).mp.	470733
22	Cardiovascular Diseases/	31817
23	(cardiovascular or cv or cvd or vascular or coronary).tw.	1824792
24	heart disease$.tw.	225534
25	cardiovascular risk factor$.tw.	56404
26	or/12-19	1083238
27	or/22-25	1941192
28	Randomized Controlled Trial/	638226
29	Clinical Trial/	988522
30	randomized controlled trial.pt. or randomised controlled trial.mp. [mp=title, abstract, heading word, drug trade name, original title, device manufacturer, drug manufacturer, device trade name, keyword, floating subheading word, candidate term word]	34253
31	controlled clinical trial/	466002
32	trial*.ti,ab.	1517240
33	pedomet*.mp.	4327
34	((step* or walk*) adj2 (count* or sensor or meter)).ti,ab.	5971

© 2021 Hodkinson A et al. *JAMA Network Open.*

35	Accelerometry/ or (accelerom* or actimeter or actigraph or actiwatch or GT3X or fitbit).ti,ab.	27313
36	((activit* or move* or motion or energy or exercise) adj2 (monitor* or sens* or detect* or count*)).tw.	87436
37	11 or 26 or 27	3397736
38	or/28-32	2324948
39	or/33-36	118012
40	37 and 38 and 39	2234
**41**	**limit 40 to (year="2000 - current")**	**2081**

© 2021 Hodkinson A et al. *JAMA Network Open.*

PsycInfo

# ▲	Searches	Results
1	Diabetes mellitus/	5321
2	diabet*.ti.	14551
3	exp Diabetes Mellitus/	8726
4	((type 2 or type ii) adj2 diabet*).ti,ab.	8188
5	((non insulin* depend* or non insulin* depend* or non-insulin?depend* or non insulin?depend*) adj1 diabet*).ti,ab.	189
6	(T2DM or T2D or TIIDM or TIID or NIDDM or MODY or MODM or AODM).ti,ab.	1650
7	((obes* or overweight) adj5 diabet*).ti,ab.	2742
8	prediabetic state.tw.	11
9	(prediabetes or pre diabetes or pre-dm or subclinical diabetic or raised glucose intolerance or impaired glucose level$ or impaired glucose tolerance or IGT or impaired fasting glucose or IFT or FPG or fasting plasma glucose or impaired glucose regulation or impaired glucose metabolism or raised glycated haemoglobin or raised glycated hemoglobin or high glycated Hb or hyperglycaemia or hyperglycemia or without diabet* or without diagnosed diabet*).tw.	4208
10	((prevent* or avoid* or delay* or decreas* or reduc*) adj2 (type II diabetes or type 2 diabetes or T2D or DM or diabetes)).ti,ab.	1202
11	or/1-10	22859
12	exp Obesity/	25230
13	Obese.tw.	16441
14	(overweight or obese or over-weight or over weight or overeating or over eating or over-eating).ti.	7144
15	exp Overweight/	26664
16	(BMI or body mass index).af.	62048
17	Weight gain/	3210
18	(Overweight or over weight or obesity or adipose).af.	136755
19	(body mass index or BMI).mp.	33283
20	exp Cardiovascular Disorders/	63480
21	(cardiovascular or cv or cvd or vascular or coronary).tw.	57571
22	heart disease$.tw.	10469
23	cardiovascular risk factor$.tw.	2354
24	or/12-19	156833
25	Randomized Controlled Trial.mp.	19510
26	randomized controlled trial.pt. or randomised controlled trial.mp. [mp=title, abstract, heading word, table of contents, key concepts, original title, tests & measures, mesh]	3827
27	trial*.ti,ab.	187204
28	pedomet*.mp.	898
29	((step* or walk*) adj2 (count* or sensor or meter)).ti,ab.	795
30	Accelerometry/ or (accelerom* or actimeter or actigraph or actiwatch or GT3X).ti,ab.	4253
31	((activit* or move* or motion or energy or exercise) adj2 (monitor* or sens* or detect* or count*)).tw.	12367
32	11 or 24	170531
33	25 or 26 or 27	187468
34	28 or 29 or 30 or 31	17347

© 2021 Hodkinson A et al. *JAMA Network Open.*

35	32 and 33 and 34	495
**36**	**limit 35 to yr="2000 -Current"**	**492**

© 2021 Hodkinson A et al. *JAMA Network Open.*

CENTRAL

# ▲	Searches	Results
1	Diabetes mellitus/	9817
2	diabet*.ti.	58382
3	exp Diabetes Mellitus/	31706
4	((type 2 or type ii) adj2 diabet*).ti,ab.	38918
5	((non insulin* depend* or non insulin* depend* or non-insulin?depend* or non insulin?depend*) adj1 diabet*).ti,ab.	1965
6	(T2DM or T2D or TIIDM or TIID or NIDDM or MODY or MODM or AODM).ti,ab.	10948
7	((obes* or overweight) adj5 diabet*).ti,ab.	6269
8	prediabetic state.tw.	31
9	(prediabetes or pre diabetes or pre-dm or subclinical diabetic or raised glucose intolerance or impaired glucose level$ or impaired glucose tolerance or IGT or impaired fasting glucose or IFT or FPG or fasting plasma glucose or impaired glucose regulation or impaired glucose metabolism or raised glycated haemoglobin or raised glycated hemoglobin or high glycated Hb or hyperglycaemia or hyperglycemia or without diabet* or without diagnosed diabet*).tw.	17285
10	((prevent* or avoid* or delay* or decreas* or reduc*) adj2 (type II diabetes or type 2 diabetes or T2D or DM or diabetes)).ti,ab.	4520
11	or/1-10	80240
12	exp Obesity/	14067
13	Obese.tw.	23680
14	(overweight or obese or over-weight or over weight or overeating or over eating or over-eating).ti.	14192
15	exp Overweight/	15901
16	(BMI or body mass index).af.	64683
17	Weight gain/	2496
18	(Overweight or over weight or obesity or adipose).af.	48579
19	(body mass index or BMI).mp.	64681
20	(cardiovascular or cv or cvd or vascular or coronary).tw.	139427
21	heart disease$.tw.	17801
22	cardiovascular risk factor$.tw.	5514
23	or/12-22	95752
24	pedomet*.mp.	1861
25	((step* or walk*) adj2 (count* or sensor or meter)).ti,ab.	1940
26	Accelerometry/ or (accelerom* or actimeter or actigraph or actiwatch or GT3X or fitbit).ti,ab.	5008
27	((activit* or move* or motion or energy or exercise) adj2 (monitor* or sens* or detect* or count*)).tw.	7415
28	11 or 23	106036
29	24 or 25 or 26 or 27	9390
30	28 and 29	1210
**31**	**limit 30 to yr="2000 - current"**	**1171**

© 2021 Hodkinson A et al. *JAMA Network Open.*

eTable 2: Citations of eligible studies for review

1. *Anderson D.R. Health Beliefs, Will to Live, Hope, and Social Support in a Pedometer-Based Exercise Intervention among Cardiac Rehabilitation Patients. 2015. Available at: https://etd.ohiolink.edu/!etd.send_file?accession=osu1434901973&disposition=inline.*
2. Alonso-Domínguez R, Patino-Alonso MC, Sánchez-Aguadero N, García-Ortiz L, Recio-Rodríguez JI, Gómez-Marcos MA. Effect of a multifactorial intervention on the increase in physical activity in subjects with type 2 diabetes mellitus: a randomized clinical trial (EMID Study). *Eur J Cardiovasc Nurs.* 2019;18(5):399-409.
3. Andrews R, Cooper A, Montgomery A, et al. Diet or diet plus physical activity versus usual care in patients with newly diagnosed type 2 diabetes: The Early ACTID randomised controlled trial. *The Lancet.* 2011;378(9786):129-139.
4. Araiza P, Hewes H, Gashetewa C, Vella CA, Burge MR. Efficacy of a pedometer-based physical activity program on parameters of diabetes control in type 2 diabetes mellitus. *Metabolism.* 2006;55(10):1382-1387.
5. Bjorgaas MR, Vik JT, Stolen T, Lydersen S, Grill V. Regular use of pedometer does not enhance beneficial outcomes in a physical activity intervention study in type 2 diabetes mellitus. *Metabolism.* 2008;57(5):605-611.
6. Chudowolska-Kiełkowska M, Małek Ł A. A nurse-led intervention to promote physical activity in sedentary older adults with cardiovascular risk factors: a randomized clinical trial (STEP-IT-UP study). *Eur J Cardiovasc Nurs.* 2020;19(7):638-645.
7. Claes J, Cornelissen V, McDermott C, et al. Feasibility, Acceptability, and Clinical Effectiveness of a Technology-Enabled Cardiac Rehabilitation Platform (Physical Activity Toward Health-I): Randomized Controlled Trial. *J Med Internet Res.* 2020;22(2):e14221.
8. Cupples ME, Dean A, Tully MA, et al. A feasibility study of a randomized controlled trial of a pedometer based exercise intervention to promote physical activity in cardiac rehabilitation. *Eur J Prev Cardiolog.* 2012;1):S29.
9. Dasgupta K, Rosenberg E, Joseph L, et al. Physician step prescription and monitoring to improve ARTERial health (SMARTER): a randomized controlled trial in patients with type 2 diabetes and hypertension. *Diabetes Obes Metab.* 2017(pagination).
10. De Greef K, Deforche B, Tudor-Locke C, De Bourdeaudhuij I. A cognitive-behavioural pedometer-based group intervention on physical activity and sedentary behaviour in individuals with type 2 diabetes. *Health Educ Res.* 2010;25(5):724-736.
11. De Greef K, Deforche B, Tudor-Locke C, De Bourdeaudhuij I. Increasing physical activity in Belgian type 2 diabetes patients: a three-arm randomized controlled trial. *Int J Behav Med.* 2011;18(3):188-198.

© 2021 Hodkinson A et al. *JAMA Network Open.*

12. De Greef KP, Deforche BI, Ruige JB, et al. The effects of a pedometer-based behavioral modification program with telephone support on physical activity and sedentary behavior in type 2 diabetes patients. *Patient Education and Counseling*. 2011;84(2):275-279.
13. Diedrich A, Munroe DJ, Romano M. Promoting physical activity for persons with diabetes. *Diabetes Educ*. 2010;36(1):132-140.
14. Engel L, Lindner H. Impact of using a pedometer on time spent walking in older adults with type 2 diabetes. *Diabetes Educ*. 2006;32(1):98-107.
15. Fayehun AF, Olowookere OO, Ogunbode AM, Adetunji AA, Esan A. Walking prescription of 10 000 steps per day in patients with type 2 diabetes mellitus: A randomised trial in Nigerian general practice. *Br J Gen Pract*. 2018;68(667):e139-e145.
16. Frederix I, Hansen D, Coninx K, et al. Medium-Term Effectiveness of a Comprehensive Internet-Based and Patient-Specific Telerehabilitation Program With Text Messaging Support for Cardiac Patients: Randomized Controlled Trial. *J Med Internet Res*. 2015;17(7):e185.
17. Greaney ML, Askew S, Wallington SF, Foley PB, Quintiliani LM, Bennett GG. The effect of a weight gain prevention intervention on moderate-vigorous physical activity among black women: the Shape Program. *Int*. 2017;14(1):139.
18. Grey EB, Thompson D, Gillison FB. Effects of a Web-Based, Evolutionary Mismatch-Framed Intervention Targeting Physical Activity and Diet: a Randomised Controlled Trial. *Int J Behav Med*. 2019;26(6):645-657.
19. Guiraud T, Granger R, Gremeaux V, et al. Telephone support oriented by accelerometric measurements enhances adherence to physical activity recommendations in noncompliant patients after a cardiac rehabilitation program. *Arch Phys Med Rehabil*. 2012;93(12):2141-2147.
20. Houle J, Doyon O, Vadeboncoeur N, Turbide G, Diaz A, Poirier P. Innovative program to increase physical activity following an acute coronary syndrome: randomized controlled trial. *Patient Educ Couns*. 2011;85(3):e237-244.
21. Houle J, Doyon O, Vadeboncoeur N, Turbide G, Diaz A, Poirier P. Effectiveness of a pedometer-based program using a socio-cognitive intervention on physical activity and quality of life in a setting of cardiac rehabilitation. *Can J Cardiol*. 2012;28(1):27-32.
22. Karstoft K, Winding K, Knudsen SH, et al. The effects of free-living interval-walking training on glycemic control, body composition, and physical fitness in type 2 diabetic patients: a randomized, controlled trial. *Diabetes Care*. 2013;36(2):228-236.
23. Katzmarzyk PT, Champagne CM, Tudor-Locke C, et al. A short-term physical activity randomized trial in the Lower Mississippi Delta. *PLoS ONE*. 2011;6(10):e26667.
24. Kirk A, Barnett J, Leese G, Mutrie N. A randomized trial investigating the 12-month changes in physical activity and health outcomes following a physical

© 2021 Hodkinson A et al. *JAMA Network Open.*

activity consultation delivered by a person or in written form in Type 2 diabetes: Time2Act. *Diabet Med.* 2009;26(3):293-301.
25. Lewis ZH, Ottenbacher KJ, Fisher SR, et al. Effect of Electronic Activity Monitors and Pedometers on Health: Results from the TAME Health Pilot Randomized Pragmatic Trial. *Int J Environ Res Public Health.* 2020;17(18).
26. Lyons EJ, Swartz MC, Lewis ZH, Martinez E, Jennings K. Feasibility and Acceptability of a Wearable Technology Physical Activity Intervention With Telephone Counseling for Mid-Aged and Older Adults: A Randomized Controlled Pilot Trial. *JMIR Mhealth Uhealth.* 2017;5(3):e28.
27. Lystrup R, Carlsen D, Sharon DJ, Crawford P. Wearable and interactive technology to share fitness goals results in weight loss but not improved diabetes outcomes. *Obes Res Clin Pract.* 2020;14(5):443-448.
28. Martin SS, Feldman DI, Blumenthal RS, et al. mActive: A Randomized Clinical Trial of an Automated mHealth Intervention for Physical Activity Promotion. *J Am Heart Assoc.* 2015;4(11):09.
29. Miyamoto T, Fukuda K, Oshima Y, Moritani T. Non-locomotive physical activity intervention using a tri-axial accelerometer reduces sedentary time in type 2 diabetes. *Phys Sportsmed.* 2017;45(3):245-251.
30. Paschali AA, Goodrick GK, Kalantzi-Azizi A, Papadatou D, Balasubramanyam A. Accelerometer feedback to promote physical activity in adults with type 2 diabetes: a pilot study. *Percept Mot Skills.* 2005;100(1):61-68.
31. Paula TP, Viana LV, Neto ATZ, Leitao CB, Gross JL, Azevedo MJ. Effects of the DASH Diet and Walking on Blood Pressure in Patients With Type 2 Diabetes and Uncontrolled Hypertension: A Randomized Controlled Trial. *J Clin Hypertens (Greenwich).* 2015;17(11):895-901.
32. Pekmezi D, Ainsworth C, Joseph RP, et al. Pilot Trial of a Home-based Physical Activity Program for African American Women. *Med Sci Sports Exerc.* 2017;49(12):2528-2536.
33. Piette JD, Richardson C, Himle J, et al. A randomized trial of telephonic counseling plus walking for depressed diabetes patients. *Med Care.* 2011;49(7):641-648.
34. Plotnikoff R, Karunamuni N, Courneya K, Sigal R, Johnson J, Johnson S. The Alberta Diabetes and Physical Activity Trial (ADAPT): A randomized trial evaluating theory-based interventions to increase physical activity in adults with type 2 diabetes. *Ann Behav Med.* 2013;45(1):45-56.
35. Silfee V, Petosa R, Laurent D, Schaub T, Focht B. Effect of a behavioral intervention on dimensions of self-regulation and physical activity among overweight and obese adults with type 2 diabetes: a pilot study. *Psychol Health Med.* 2016;21(6):715-723.
36. Tudor-Locke C, Bell R, Myers A, et al. Controlled outcome evaluation of the First Step Program: A daily physical activity intervention for individuals with type II diabetes. *Int J Obes (Lond).* 2004;28(1):113-119.

© 2021 Hodkinson A et al. *JAMA Network Open.*

37. Van Dyck D, De Greef K, Deforche B, et al. The relationship between changes in steps/day and health outcomes after a pedometer-based physical activity intervention with telephone support in type 2 diabetes patients. *Health Educ Res*. 2013;28(3):539-545.
38. Yates T, Davies M, Gorely T, Bull F, Khunti K. Effectiveness of a pragmatic education program designed to promote walking activity in individuals with impaired glucose tolerance: a randomized controlled trial. *Diabetes Care*. 2009;32(8):1404-1410.
39. Yates T, Edwardson CL, Henson J, et al. Walking Away from Type 2 diabetes: a cluster randomized controlled trial. *Diabet Med*. 2017;34(5):698-707.

© 2021 Hodkinson A et al. *JAMA Network Open.*

eTable 3: Summary of intervention characteristics by study

**Study author**	**Aims of intervention**	**Theoretical approach**	**Intervention components**	**Control components**	**Delivery method (including format and provider)**	**Number of sessions (time of each session)**	**Length of interventio n (weeks)**	**Goals setting and uptake**
**Pedometers**								
Alonso-Dominguez 2019	To assess the effect of a multifactorial intervention approach based on a smartphone app, walking, and a diet workshop on physical activity in subjects with T2DM	NA	Counselling on physical activity was provided for all participants for 5 minutes and included advice on compliance with the current international recommendatio ns (to walk at least 10,000 steps daily and avoid a sedentary lifestyle).<br><br>Physical activity was objectively recorded for Seven consecutive days using a	Participants were given a leaflet as support	All study participants received standardised counselling for 10 minutes on physical activity and a healthy diet combined (5 mins physical activity and 5 mins on diet)	10 mins counselling	3 months	10,000 steps a day<br><br>NR

© 2021 Hodkinson A et al. *JAMA Network Open.*

			digital pedometer with two piezoelectric sensors (Omron HJ-321 Tri-axis) placed on the right side of the waist.					
Anderson 2015	To evaluate the effect of pedometer tracking on exercise adherence among post-Cardiac rehabilitation patients in a randomized study with control participants engaging in usual care	NA	Pedometer worn daily with logbook use to record steps	NR	Unsupervised pedometer tracking after CR is complete. By specialist staff	1 at the beginning of the study	12	NA<br><br>NR
Andrews 2011	To investigate the effects of diet and physical activity on blood pressure and glucose concentrations	NA	Each patient was given a pedometer (Digiwalker CW200, Yamax, Japan) and a folder containing	Usual care consisted of standard dietary and exercise advice after randomisation and at the end of the	The diet was not prescriptive; goals were negotiated individually with each	Dietary advice and goal setting was reinforced by nine 30 min appointment	6 months	NR<br><br>98%

© 2021 Hodkinson A et al. *JAMA Network Open.*

			motivating literature and pages for recording daily physical activity (pedometer readings)	study, with reviews by a study doctor and nurse at baseline and at 6 and 12 months. This group was used as the control.	participant during their first session with the dietitian and were reviewed at each visit. Participants saw the dietitian for 1 h at randomisation and for 30 min at each of 3, 6, 9, and 12 months.	s with study nurses—roughly one every 6 weeks over the course of the study.		
Araiza 2006	Determine whether a recommendation to accumulate 10,000 steps per day would result in significant improvements in parameters of glycaemic control, insulin sensitivity, cardiovascular risk, and oxidative stress in sedentary	NA	Each participant wore a pedometer throughout the day and was trained how to use it. Pedometers were positioned on the waist. Steps were recorded in an activity log.	Instructed to maintain their normal activity habits throughout the 6-week intervention	In active group participants were instructed to walk 10,000 steps on 5 or more days of the week for 6 weeks.	NA - self monitored	6	NA<br><br>NR

© 2021 Hodkinson A et al. *JAMA Network Open.*

	patients with type 2 diabetes							
Bjorgaas 2008	Determine whether regular used of pedometers increases walking and/or enhances health related beneficial effect in type 2 diabetic participants	NA	Pedometer and logbook 3 weekdays twice per month	Participants were encouraged to increase the average daily time spent walking from one visit to another, guided by logbook	Participants meet FTF with study nurse baseline, 1 month, 3 months and 6 months	NR	26	Goals were set at every nurse meeting after baseline<br><br>68% completion rate amongst participants
Chudowolsk a-Kielkowska 2020	A randomized clinical trial of a nurse-led intervention that included goal setting and phone support to increase physical activity in sedentary older adults with cardiovascular risk factor in primary care setting compared to standard of care	NA	All randomized subjects received a simple pedometer (M2 Smart band, Enwei Technology Co. Ltd., Shenzhen, China) and were told to note the number of steps per day in a handed notebook. Subjects in the study group	Usual care with no step goal or supportive calls	10-min nurse-led tutorial with distributed hand outs on health benefits of regular physical activity	1 session with nurse	3 months	Minimum 7,000 steps per day

© 2021 Hodkinson A et al. *JAMA Network Open.*

			were told to achieve a goal of minimum 7000 steps per day, while the control group did not have a set goal and was just told to note the number of steps per day					
Cupples 2013	Examine the use of pedometer step count goals to promote physical activity for cardiac rehabilitation patients	NA	Pedometer, dairy to record step counts with feedback from facilitator	Facilitator recorded baseline pedometer data, but no feedback information was given	Face-to-face or by telephone, facilitator contact made weekly	Weekly contact with the facilitator	6	Gradual 10% increase in average daily count aiming for 10,000 steps/day<br><br>93% completion rate
Dasgupta 2017	Impact of intervention on physical activity, but also gauge biological effects by evaluating several cardio-metabolic measures	NA	To achieve a net increase over baseline of 3000 steps/day over 1 year using a pedometer, step count log and step count prescription	Received advice to engage in 30 to 60 mins of activity daily, consistent with usual care	Participants were typically seen by their physician in a clinical setting 3-4 times over a 12-15-month period.	3-4 times over a 12-15-month period. Time per session unclear for intervention group, in control group they were	52	3000 steps/day increase over 1 year<br><br>79% completion rate

© 2021 Hodkinson A et al. *JAMA Network Open.*

						engaged in 30-40 mins of activity		
De Greef 2010	Investigate the benefits of a pedometer and a cognitive behavioural intervention for promoting PA in type 2 diabetes patients	Cognitive-behavioural therapy	Received pedometer and a pedometer diary during intervention as motivational tools.	Received usual care from their endocrinologist and a single education session about type 2 diabetes and PA which was the same as the first session of the intervention group	Group meetings which involved motivational interviewing, then an implementation plan was developed with coaches.	90-min group meetings, first three given every 2 weeks, the last two sessions were given over interval of 3 and 4 weeks.	12	At each session coaches set new goals<br><br>75% compliance rate
De Greef 2011 (1)	To promote PA and decreasing sedentary behaviour	Cognitive-behavioural therapy, the diabetes prevention program, the first step program and motivational interviewing	face-to-face session, a pedometer and telephone support	No intervention	face-to-face sessions and telephone support	Seven call into total and one interview	24	>10,000 steps/day<br><br>96% completion rate
De Greef 2011 (2)	Investigate whether a 12-week pedometer-based PA intervention delivered by a	NA	Pedometer and dairy to keep log of type of PA, duration and number of steps/days	No intervention, only received general care from GP	face-to-face delivery by GP or behaviour expert	90-min group counselling sessions over a 12-week period 9one session every 3 weeks).	12	NA<br><br>NR

© 2021 Hodkinson A et al. *JAMA Network Open.*

	trained GP individually can be as effective as group delivery by behavioural expert					Participant in the GP delivered group received three 15-min FTF consultations		
Diedrich 2010	To see whether the self-help Manpo-Kei program can be a solution to promoting exercise in people with diabetes without adding significant content and activities to a diabetes self-management education (DSME) program	NR	Received a copy of the Manpo-Kei (guide to steps) book and concise handout summarizing the key points of the book and a pedometer	Attended usual DSME programs only	Initial FTF assessment by certified diabetes educators prior to attending first DSME program session. Questionnaires mailed out at 3 months. Telephone calls also made to participants to set up the follow-up appointment	DSME includes a 2 -hour assessment and 8 hours of group classes	13	NA<br><br>62% completion rate
Engel 2006	Investigate the impact of using a pedometer on time spent walking	NR	Pedometer and exercise log to record the number of steps each day	Health-related coaching including behaviour change	6 face-to-face visits or contacts by facilitator during the 6	6 visits each the time varied	26	6000-8500 steps/d for healthy older adults and

				strategies to improve self-efficiency like goal setting	months of study			3500-5500 steps/d for older adults with chronic illnesses were considered as goals
Fayehun 2018	To evaluate whether 10,000 steps per day is believed to be a reasonable estimate of daily activity for healthy adults	NA	Pedometer and manual record for recording daily step counts	Continued with typical daily activities	Face-to-face and telephone	Face-to-face counselling each week until they reached 10,000 steps per day, telephone follow-up was also given at weeks 2, 6 and 10.	10	To accumulate 10,000 steps per day for 10 weeks.<br><br>85% completion rate
Greaney 2017	Examine the impact of the shape program, a weight gain prevention program designed for black overweight or obese women	Social cognitive theory with self-efficacy as primary mediator	Individuals randomized received, a) tailored behaviour change goals to promote the prevention of weight gain, skills training materials,	Participants were mailed semi-annual newsletters during the intervention period. The newsletter covered general wellness topics but not PA,	Coaching calls and motivational interviewing. Printed skills training material were provided to participants with assigned behaviour	Measurements taken at baseline and 12 months. Sessions of PA were summaries into 1- and 10-min bouts of MVPA	52	Tailored behaviour change goals and step goals<br><br>62% completion rate

© 2021 Hodkinson A et al. *JAMA Network Open.*

	living in the rural South		weekly interactive voice response (IVR) telephone calls for self-monitoring, monthly telephone coaching from a registered dietitian and a no-cost 12 month membership to a YMCA facility of their choice	nutrition of weight	r changes goals.			
Grey 2019	To assess the effectiveness of the 12-week evolutionary mismatch-framed, self-directed intervention in increasing PAL and reducing EI. We also examined whether any changes in activity or diet achieved by the intervention	BCT (Taxonomy v.1, 2013)	The intervention (“Evolife”) was based around a website that aimed to provide participants with information, framed from an evolutionary mismatch perspective, about physical activity and healthy eating, and	Manual goals were set by participant individually.	Intervention websites provide all relevant intervention information	The researcher gave participants a multisensory physical activity monitor to wear for 7 days following the assessment visit, along with a 3-day food diary and set of kitchen	12 weeks	Goals set for behaviour change<br><br>94-97% completion rate

© 2021 Hodkinson A et al. *JAMA Network Open.*

	were sufficient to generate clinically meaningful changes in metabolic control and/or anthropometric risk markers for developing type 2 diabetes and cardiovascular disorder		advice on how to make behavioural changes to improve health. One of the goals was a daily step goal and to help with this, participants were given a pedometer.			scales to also complete over the following week. Full instructions on both the activity monitor and food diary were explained by the researcher and given in printed form for the participants to take away. Activity monitors and completed food diaries were collected by the researcher approximatel y 8 days after each assessment (i.e.		

© 2021 Hodkinson A et al. *JAMA Network Open.*

						when the activity monitoring had been completed)		
Houle 2011, 2012	To evaluate the impact of a home-based cardiac rehabilitation program led by a clinical nurse specialist on PA behaviour at 3, 6, 9 and 12 months after an acute coronary syndrome	Social cognitive theory framework	Participants given pedometer (Yamax Digiwalker SW-200), diary and information regarding PA after an acute coronary syndrome	Provided with standard advice on PA at discharge	Intervention delivered FTF by clinical nurse specialist before hospital discharge	Follow-up sessions included one phone call within 2 weeks after discharge and 5 FTF consultations (at 6 weeks and 3, 6, 9 and 12 months after the event). Sessions lasted between 30-60 mins	52	Intervention includes a goal setting element with target > 3,000 steps/d at baseline<br><br>69% completed the trial
Katzmarzyk 2011	To assess whether a pedometer-based educational intervention could increase MVPA in short term and to assess whether	NA	Received the same educational materials, but were given the YAMAX Digi-Walker SW-200 pedometer to record steps	Education group only received a brochure detailing the importance of physical activity for maintaining health and guidance on	Self-monitored daily with instructions to engage in usual activity then to increase. Facilitator not involved in	Participants recorded their daily steps in a log sheet	2	NA<br><br>80% completion rate

© 2021 Hodkinson A et al. *JAMA Network Open.*

	change in steps/day is associated with change in MVPA			how to increase physical activity	delivery of intervention			
Kirk 2009	To assess whether those randomised to PA consultation delivered by a person or in written form increase PA levels over 6 and 12 months	Transtheoretical model of behaviour change	Self-instructional workbook included a pedometer	Received a two-page information leaflet by Tayside Diabetes network.	FTF consultation, workbook, follow-up phone calls	30 min FTF consultation with trained researcher at baseline and 6 months	52	NA<br><br>87% completion rate
Lewis 2020	The TAME health (Testing Activity Monitors' Effect on health) pilot randomized controlled trial aimed to investigate a low intensity intervention to increase PA and decrease cardiovascular disease risk within the primary care setting	Behavioural change techniques including goal setting on behaviour and health outcome, providing instructions and information on consequences, as well as facilitating social support	Participants in the pedometer group were given a digital pedometer (Digi-walker CW-700/701, YAMAX, San Antonio, TX, USA) and a PA log to record their daily steps, activity time, and distance walked.	Participants in the EAM group were given an UP24 monitor by Jawbone (San Francisco, CA, USA) and downloaded the corresponding UP app on their personal smartphones	All participants received a brief 5 A's counselling consisting of components: assess, advise, agree, assist, and arrange.	FTF consultation with regular meeting over 12 weeks period	12	Goal setting could be used but was not specifically part of intervention<br><br>100% completion

© 2021 Hodkinson A et al. *JAMA Network Open.*

Paula 2015	To evaluate the effect of the DASH diet associated with increased walking on ABPM in patients with type 2 diabetes and uncontrolled hypertension	NA	Patients were asked to increase physical activity by walking at least 15 to 20 minutes per day, 5 days per week, in addition to their baseline activities. A pedometer was provided to be used during the 4-week intervention period. During the study, twice a week, text messages (SMS) were sent or phone calls were made to stimulate compliance with the general protocol. Patient counselling on diet was performed	Control Group. Patients received dietary recommendations according to American Diabetes Association guidelines 6 and were instructed to maintain their usual physical activity during the study. A pedometer was provided to be used every day exclusively in the first and last week of the study to record usual PA	At baseline, patients underwent 24-hour ABPM and their usual walking habits were assessed by daily step count using a pedometer during 1week.Clinical, nutritional, and physical activity were assessed and laboratory evaluations were performed. This run-in period involved two office visits. The duration of the trial was 4 weeks and at the end	FTF consultation with two office visits	4 weeks	NA<br><br>100%

© 2021 Hodkinson A et al. *JAMA Network Open.*

			by the research dietitian (TPP) and on physical activity by the physical educator (AZN).		of the study, ABPM and anthropometric and laboratory measurements were performed. Patients were advised not to change any usual medication during the study.			
Pekmezi 2017	To assess whether women assigned to the Home-based, individually tailored Physical activity Print (HIPP) intervention would more greatly increase PA and related psychosocial variables at 6 months when compared with the control group	Social cognitive theory and the transtheoretical model	Accusplit pedometers and activity logs provided to encourage self-monitoring of exercise behaviour.	Received mailings with cancer prevention information on topics other than PA. These were received at time points identical to those of the intervention group	For self-reported data participants wore accelerometers on their hip for 7 consecutive days at baseline and 6 months (contact with trial investigators). Accelerometers were mailed to participants	Accelerometer was always worn over the 7 days from baseline. Contact time at 6 months not reported	26	Goal setting used to measure self-regulation nature of intervention<br><br>61% returned at least three of the four updated surveys

© 2021 Hodkinson A et al. *JAMA Network Open.*

Piette 2011	Evaluate the impact of telephone-delivered cognitive behavioural therapy (CBT) targeting patients' management of depressive symptoms, physical activity levels, and diabetes-related outcomes	CBT focused on depressive symptoms and links between depression, PA and diabetes outcomes	Intervention patients participated in a 12-month telephone-delivered CBT program.<br><br>Six weeks after completing their baseline assessment, all patients were sent an Omron HJ-720 ITC pedometer with a built-in clock and electronic memory. Pedometers were sent blinded using a removable sticker, and patients were instructed to wear the pedometer throughout walking hours for seven consecutive days.	Usual care patients received: a copy of the Feeling Good Handbook -a self-help book based on cognitive behavioural therapy for depression.	Telephone delivered	The CBT program included an initial intensive phase of 12-weekly sessions followed by nine monthly booster sessions. counsellors introduced concepts related to a pedometer-based walking program.	12 months	Patient manuals included logs that they could use to complete CBT homework exercises and to monitor their progress toward step-count goals<br><br>86%

© 2021 Hodkinson A et al. *JAMA Network Open.*

Plotnikoff 2013	Explore the effectiveness of two innovative/ theoretically based behavioural-change strategies to increase PA and reduce haemoglobin A1c (A1c) in T2DM adults	the Theory of Planned Behaviour, the Health Belief Model, Protection Motivation Theory, Social Cognitive Theory, and the Transtheoretical Model to predict forward PA behaviour stage of change transitions	Group 2: Participants in this group received a pedometer, logbook, and calendar to chart their progress. (There was no specific intervention recommendation regarding steps per week.)<br><br>Group 3: Addition of telephone counselling	Group 1: received standard print PA educational materials provided by the Canadian Diabetes Association (i.e., control group).	Mixed FTF (Grp 2) and telephone counselling (Grp 3)	Grp 2: The materials were tailored to be season specific (i.e., winter, spring, summer and autumn versions) and were mailed every 3 months for 12 months. Study participants completed a stage measure at baseline, 3, 6 and 9 months<br><br>Grp 3: counselling was carried out by five individuals with relevant degree qualifications related	18 months	NA<br><br>74% provided PA data at 18 months

© 2021 Hodkinson A et al. *JAMA Network Open.*

						to PA promotion and/or counselling and who received a 1-day, 7-h training course		
Silfee 2016	To determine the preliminary effect of a behavioural intervention on the use of self-regulation strategies and moderate-to-vigorous physical activity (MVPA) in overweight and obese adults with type 2 diabetes	behavioural intervention that included goal setting, time management, and enlisting social support to help participants plan their weekly PA.	Participants were given weekly PA logs and pedometers to self-monitor their PA for two weeks. The control group received information regarding their measured PA habits.<br><br>At session three, both groups received the BodyMedia armband to wear for seven days.<br>The intervention group reviewed behavioral goals	The control group received written recommendations for PA for adults with T2DM, and a pedometer	FTF training 6 weeks prior to intervention was provided. Then during the intervention, they met individually with a researcher in person.	Both groups met individually with a researcher four times over five weeks.<br><br>The first and second visits were one week apart. Visit three was scheduled two weeks after visit two.<br><br>The third and fourth visits were one week apart.	7 days	PA goals were set.<br><br>88%

© 2021 Hodkinson A et al. *JAMA Network Open.*

			and identified barriers to PA.<br><br>They set PA goals and self-monitored their PA.<br>The final visit had both groups completing the post-test self-regulation measurement and receiving information regarding their PA.			During the first visit, both groups completed self-regulation measurements and received a BodyMedia armband to wear for seven days.		
Tudor-Locke 2004	To assess if first step program is associate with improvements in physical activity (steps per day) and whether increased physical activity was related to improvements in cardiovascular health, glycaemic	Program based on theoretical principles of self-efficacy and social support	Pedometers provided and the program manual containing goal setting and problem-solving exercises, as well as calendars for self-monitoring steps/day	Received postcards thanking participants for taking part in the study.	Face-to-face assessments at meeting	Initial 4 weeks participants were asked to attend four weekly group meetings, remaining 12 weeks participants asked to use pedometers and calendars for goal	16	Increase PA > 3000 steps/day.<br><br>78% completion rate

© 2021 Hodkinson A et al. *JAMA Network Open.*

	control, and lipid profiles					setting and self-monitoring		
Van Dyck 2013	Examine the effects of physical activity program were mediated by theoretical constructs targeted by the intervention, both post-intervention and at 1 year	Intervention based on self-determination theory and the transtheoretical model	Accelerometer, pedometer and IPAQ. Pedometer and accelerometer were worn at the waist during waking hours for 7 days. Activity log was used to record step taken and the type duration of walking activities	Unclear	Face-to-face session, pedometer use and seven phone calls (tailored motivational interviewing)	Telephone calls ranged from 15 to 20 min spread over a 24-week period	24	NA<br><br>96% completion rate
Yates 2009	Investigate whether a pragmatic structured education program with and without pedometer use is effective for promoting physical activity and improving glucose tolerance	NA	Group 1 received a 3-h group-based structured education program designed to promote walking activity using personalized steps per-day goals and pedometers.	Group 3 received a brief information leaflet (control condition)	FTF	A single-session group based education program. The program is 180 min long; 105 min are dedicated to addressing the causes, complications, timeline, and identity	12 months	Sedentary participants were encouraged to increase their activity levels by at least 3,000 steps per day, equivalent to ~30 min of walking.<br><br>82%

© 2021 Hodkinson A et al. *JAMA Network Open.*

	in those with impaired glucose tolerance (IGT)		Group 2 received a 3-h group-based structured education program designed to promote walking activity using generic time-based goals.			of IGT and 75 min are targeted to addressing the perceived effectiveness of exercise as a treatment for IGT, walking self-efficacy beliefs, barriers to walking, and self-regulatory strategies		
Yates 2017	To investigate whether an established behavioural intervention, Walking Away from Type 2 diabetes mellitus, is effective at promoting and sustaining increased walking activity when delivered	Protection motivation theory in which an association between perceived disease severity and the intention to be physically active in those with Type 2 diabetes mellitus has been demonstrated	Walking Away from Type 2 diabetes mellitus, a pragmatic 3-h group-based structured education programme incorporating pedometer use with annual follow-on refresher sessions were	Control participants received a standardized booklet detailing information on Type 2 diabetes mellitus risk informed by Leventhal's common-sense model and how physical activity and lifestyle change can be	The participants in the intervention group were provided with pedometer and step/day diary provided free. They were encouraged to increase their physical activity levels up to 3000	2 follow up session: 12 and 24 months	52	Increasing PA by 500 steps/day every fortnight<br><br>71% completion rate

© 2021 Hodkinson A et al. *JAMA Network Open.*

	within primary care.		offered to the participants	used to prevent or delay the disease	step/day over baseline levels depending on individual preference and ability. Participants set an action plan detailing where, when and how their first proximal goal would be reached and were encouraged to repeat this process for each new goal.			
**Accelerometer/Fitbits**								
Claes 2020	To assess the feasibility, acceptance, and short-term clinical effectiveness of the PATHway system for maintaining PA and physical fitness	PATHway system involved behaviour change goal	Patients were provided with accelerometer	Usual care	PATHway system installed in participants home	Participants were instructed to wear the Actigraph GT9X Link on the nondominant wrist for 24 h per day during 7-consecutive	6 months	150 min of moderate intensity PA per week.<br><br>83% completion

© 2021 Hodkinson A et al. *JAMA Network Open.*

	of patients with CVD after completion of an ambulatory centre-based CR program					days.		
Frederix 2015	To assess medium-term effectiveness of a patient-specific, comprehensive cardiac tele-rehabilitation program in addition to standard ambulatory cardiac rehabilitation	NA	Yorbody accelerometer motion sensor used, internet-based tele-rehabilitation in addition to Centre-based rehabilitation	Centre-based rehabilitation alone	Psychologist aimed to improve patient self-efficiency to change prior lifestyle. SMS message sent out to provide motivational content	At least 2 exercise training sessions per week. Patients instructed to exercise for 45 to 60 mins per session	24	Predefined exercise training goal disseminated via SMS<br><br>90% complete the study as planned
Guiraud 2012	Assess the efficacy of a strategy, based on telephone support oriented by accelerometer measurements, on the adherence to PA recommendations in cardiac patients not	NR	Accelerometer used to measure all PA outcomes	PA measured with accelerometer during the 8th week of testing period	PA measurements were recorded during a period of 2 months. Kinesiologist insisted on the importance of wearing the accelerometer. Each session	Patients participated in 45-minute fitness, gymnastics, relaxation, Qi Gong, or aquatic training sessions.	8	Goal setting for 2 weeks for EE outcome<br><br>69% of participants complete the trial

© 2021 Hodkinson A et al. *JAMA Network Open.*

	achieving PA recommendations				was monitored by a physiotherapist or kinesiologist and supervised by a cardiologist			
Karstoft 2013	Evaluate the feasibility of free-living walking training in type 2 diabetic patients and to investigate the effects of interval-walking training versus continuous-walking training upon physical fitness, body composition	NA	Interventions All subjects received a JDMate, which was worn as a pedometer throughout the study.<br><br>Subjects randomized to a training group used the JD Mate's training function, which, based on triaxial accelerometery, estimates training energy expenditure.	Subjects in the CON group were instructed to continue their habitual lifestyle for 4 months and had their JD Mate pedometer data uploaded monthly	FTF training sessions	Training groups were prescribed five sessions per week (60 min/session) and were controlled with an accelerometer and a heart-rate monitor	4 months	NA<br><br>NR

© 2021 Hodkinson A et al. *JAMA Network Open.*

Lyons 2017	To determine the feasibility, acceptability, and effect on physical activity of an intervention combining a wearable physical activity monitor, tablet device, and telephone counselling among adults aged 55-79 years	App based on behaviour change techniques and adherence to theory-based recommendations	Intervention group were lent a mini tablet mobile device (Apple iPad Mini, Apple Inc, Cupertino, CA) and a wearable electronic activity monitor (Up24, Jawbone Inc, San Francisco, CA) for home use during the study.	The wait-list control group did not receive any intervention until after their final assessment when they were provided the intervention in full	Self-monitoring but also some counselling sessions	Participants attended 4 scheduled visits of 15-20 mins	12 weeks	Goals were negotiated between the counsellors and individuals, with counsellors suggesting at least 7000 steps per day<br><br>100%
Lystrup 2020	Investigate the effects of adding virtual activity groups to a multicomponent ambulatory activity monitoring intervention in managing chronic conditions such as obesity and type 2 diabetes.	NA	The Fitbit + Friends group served as the intervention group. Subjects in the Fitbit + Friends group were assigned to a virtual fitness group with 9 other individuals from their randomized block. Every subject in the	SoloFitbit subjects with the same secure Fitbit® and WhatsApp® platforms but did not assign them to any virtual sup-port groups. Their step counts were visible only to themselves and the research coordinator who	Subjects had in-person follow up sessions at 3 and 6 months with a research coordinator	Research coordinator provided individual feedback	6 months	Each participant either stated a specific step-count goal or were recommended to reach 10,000

© 2021 Hodkinson A et al. *JAMA Network Open.*

			Fitbit + Friends group was a virtual “friend” with the rest of the group and was able to see a “leader board” which tracked individual step counts and ranked them in order. Within their smartphone applications, Fitbit + Friends subjects were able to see in real time their daily step counts in comparison with the rest of their group on a ranking board.	gave identical scripted feedback to both groups.				
Matrin 2015	To investigate whether a fully automated mobile health (mHealth) intervention with tracking	Smart texts reflected behaviour change theories	Participants used their own smartphones.<br><br>Digital physical activity tracking was performed	Control participants were blinded to the device interface	Automated smart text system	Smart texts provided smartphone-delivered coaching 3 times/day	1 week	10 000 steps/day goal<br><br>100%

© 2021 Hodkinson A et al. *JAMA Network Open.*

	and texting components would increase physical activity		using the Fitbug Orb (Chicago, IL) (Figure S1), a wearable, display-free, triaxial accelerometer that pairs with low-energy Bluetooth with compatible smartphones.					
Miyamoto 2017	Whether the use of tri-axial accelerometer can reduce sedentary time and increase non locomotive physical activity (LPA) and to investigate the effect of this intervention on parameters of glucose and fat metabolism in type 2 diabetes	NA	All participants wore a tri-axial accelerometer during intervention period and were given verbal instruction regarding their objectives	No instruction given in control group regarding physical activity, they wore an accelerometer but display was turned off so they could not receive any visual feedback	Visual feedback given to intervention groups and encouragement was provided by physical therapist to increase PA	Face-to-face meeting at start of intervention and at 4- and 8- week follow-up examinations. Time at meetings unreported	12	NA<br><br>97% completion rate
Paschali 2005	To assess whether giving activity feedback to obese,	NA	Home-based intervention where participants received a	Counselling session has the same structure as intervention, but review of	Participant received a manual at start of intervention.	8 counselling sessions, length not reported	12	NA<br><br>NR

© 2021 Hodkinson A et al. *JAMA Network Open.*

	sedentary adults with type 2 diabetes would improve their adherence to a home-based walking program		manual containing instructions on self-regulation of exercise intensity and on behavioural self-management. Focus on accelerometer data which was processed by computer	the past months exercise relied upon data in the subject diary	Individual counselling sessions with behaviour therapist structured sessions we provided			

NA: non-applicable; NR: not reported

© 2021 Hodkinson A et al. *JAMA Network Open.*

eTable 4: Risk of bias study-by-study summary and overall by each domain

Study	Random sequence generation	Allocation concealment	Blinding of outcome assessment	Incomplete outcome data	Selective reporting
Alonso-Dominguez 2019	LOW	LOW	LOW	LOW	LOW
Anderson 2015	UNCLEAR	UNCLEAR	HIGH	LOW	LOW
Andrews 2011	LOW	LOW	HIGH	LOW	LOW
Araiza 2006	UNCLEAR	UNCLEAR	UNCLEAR	UNCLEAR	UNCLEAR
Bjorgaas 2008	UNCLEAR	UNCLEAR	LOW	HIGH	LOW
Chudowolska-Kielkowska 2020	LOW	LOW	LOW	HIGH	LOW
Claes 2020	LOW	LOW	HIGH	LOW	LOW
Cupples 2013	LOW	LOW	LOW	LOW	UNCLEAR
Dasgupta 2017	LOW	UNCLEAR	LOW	LOW	LOW
De Greef 2010	LOW	LOW	LOW	LOW	LOW
De Greef 2011 (1)	UNCLEAR	UNCLEAR	LOW	LOW	LOW
De Greef 2011 (2)	LOW	LOW	LOW	LOW	LOW
Diedrich 2010	UNCLEAR	UNCLEAR	HIGH	HIGH	LOW
Engel 2006	UNCLEAR	UNCLEAR	LOW	LOW	HIGH
Feyehun 2018	LOW	LOW	HIGH	HIGH	UNCLEAR
Frederix 2015	LOW	HIGH	LOW	LOW	LOW
Greaney 2017	UNCLEAR	UNCLEAR	UNCLEAR	HIGH	UNCLEAR
Grey 2019	UNCLEAR	LOW	HIGH	LOW	LOW
Guiraud 2012	UNCLEAR	UNCLEAR	UNCLEAR	LOW	LOW
Houle 2011/12	LOW	UNCLEAR	LOW	LOW	LOW
Karstoft 2013	UNCLEAR	UNCLEAR	LOW	UNCLEAR	LOW
Katzmarzyk 2011	LOW	LOW	UNCLEAR	LOW	LOW
Kirk 2009	LOW	LOW	LOW	LOW	LOW
Lewis 2020	UNCLEAR	UNCLEAR	HIGH	LOW	UNCLEAR
Lyons 2017	LOW	LOW	HIGH	LOW	HIGH
Lystrup 2020	UNCLEAR	LOW	HIGH	LOW	HIGH
Martin 2015	LOW	UNCLEAR	LOW	LOW	LOW
Miyamoto 2017	LOW	UNCLEAR	UNCLEAR	LOW	LOW
Paschali 2005	LOW	UNCLEAR	HIGH	UNCLEAR	LOW
Paula 2015	UNCLEAR	LOW	UNCLEAR	LOW	LOW
Pekmezi 2017	LOW	UNCLEAR	UNCLEAR	LOW	LOW
Piette 2011	LOW	LOW	HIGH	LOW	LOW
Plotnikoff 2013	UNCLEAR	LOW	UNCLEAR	HIGH	LOW
Silfee 2016	UNCLEAR	LOW	HIGH	HIGH	UNCLEAR

© 2021 Hodkinson A et al. *JAMA Network Open.*

Tudor-Locke 2004	UNCLEAR	UNCLEAR	UNCLEAR	HIGH	LOW
Van Dyck 2013	LOW	LOW	LOW	LOW	LOW
Yates 2009	LOW	LOW	UNCLEAR	LOW	LOW
Yates 2017	LOW	LOW	LOW	LOW	LOW

Proportions by each judgement across all studies

Judgement	Random sequence generation	Allocation concealment	Blinding of outcome assessment	Incomplete outcome data	Selective reporting
Low	22 (58%)	20 (52%)	16 (42%)	27 (71%)	29 (76%)
High	0 (0%)	1 (3%)	12 (32%)	8 (21%)	3 (8%)
Unclear	16 (42%)	17 (45%)	10 (26%)	3 (8%)	6 (16%)

© 2021 Hodkinson A et al. *JAMA Network Open.*
